# Genome-wide association mapping revealed syntenic loci *QFhb-4AL* and *QFhb-5DL* for *Fusarium* head blight resistance in common wheat (*Triticum aestivum* L.)

**DOI:** 10.1186/s12870-019-2177-0

**Published:** 2020-01-20

**Authors:** Wenjing Hu, Derong Gao, Hongya Wu, Jian Liu, Chunmei Zhang, Junchan Wang, Zhengning Jiang, Yeyu Liu, Dongsheng Li, Yong Zhang, Chengbin Lu

**Affiliations:** 1 Institute of Agricultural Sciences for Lixiahe Region in Jiangsu, Yangzhou, 225007 China; 2 Key Laboratory of Wheat Biology and Genetic Improvement for Low & Middle Yangtze Valley, Ministry of Agriculture and Rural Affairs, Yangzhou, China; 3 Jiangsu Key Laboratory of Crop Genomics and Molecular Breeding, Yangzhou, China; 4 Collaborative Innovation Center of Henan Grain Crops, Henan Agricultural University, Zhengzhou, 45002 Henan China

**Keywords:** *Triticum aestivum* L, *Fusarium* head blight (FHB); mixed linear model (MLM), Genome-wide association study (GWAS), Single nucleotide polymorphism (SNP)

## Abstract

**Background:**

*Fusarium* head blight (FHB), primarily caused by *Fusarium graminearum, is a major threat to wheat production and* food security worldwide*.* Breeding stably and durably resistant cultivars is the most effective approach for managing and controlling the disease. The success of FHB resistance breeding relies on identification of an effective resistant germplasm. We conducted a genome-wide association study (GWAS) using the high-density wheat 90 K single nucleotide polymorphism (SNP) assays to better understand the genetic basis of FHB resistance in natural population and identify associated molecular markers.

**Results:**

The resistance to FHB fungal spread along the rachis (Type II resistance) was evaluated on 171 wheat cultivars in the 2016–2017 (abbr. as 2017) and 2017–2018 (abbr. as 2018) growing seasons. Using Illumina Infinum iSelect 90 K SNP genotyping data, a genome-wide association study (GWAS) identified 26 loci (88 marker-trait associations), which explained 6.65–14.18% of the phenotypic variances. The associated loci distributed across all chromosomes except 2D, 6A, 6D and 7D, with those on chromosomes 1B, 4A, 5D and 7A being detected in both years. New loci for Type II resistance were found on syntenic genomic regions of chromsome 4AL (*QFhb-4AL*, 621.85–622.24 Mb) and chromosome 5DL (*QFhb-5DL*, 546.09–547.27 Mb) which showed high collinearity in gene content and order. SNP markers *wsnp_JD_c4438_5568170* and *wsnp_CAP11_c209_198467* of 5D, reported previously linked to a soil-borne wheat mosaic virus (SBWMV) resistance gene, were also associated with FHB resistance in this study.

**Conclusion:**

The syntenic FHB resistant loci and associated SNP markers identified in this study are valuable for FHB resistance breeding via marker-assisted selection.

## Background

Common wheat (*Triticum aestivum* L.) is one of the most important cereals in the world and is the raw material for breads, biscuits, noodles and cakes [1]. *Fusarium* head blight (FHB), caused by *Fusarium graminearum*, is one of the most destructive fungal diseases in wheat, which spreads considerably due to farming practices and climate changes [2]. FHB does not only reduce grain yield and quality but also leads to infected kernels with excessive deoxynivalenol (DON), resulting in severe harm to human and animal health [3]. China has the largest wheat production and consumption suffering from severe FHB damages, especially in the Middle and Lower Reaches of the Yangtze River with its warm, humid environment. In recent years, FHB has become more serious and expanded in the major wheat production area of the Yellow and Huai River Valleys [4].

The most effective way for wheat producers to manage and control FHB is by breeding resistant cultivars. Great efforts have been made to find FHB resistance genes and understand the genetic mechanism of the resistance [5–9]. The genetic mechanisms for FHB resistance are complex, and the genotype by environment interaction has very strong effects on trait expression [10, 11]. Resistance to *F. graminearum* in wheat has been classified into five categories: (1) type I for resistance to initial infection by the pathogen, (2) type II for resistance to fungal spread along the rachis, (3) type III for resistance to kernel infection, (4) type IV for resistance to toxin accumulation, and (5) type V for tolerance [12, 13]. Many quantitative trait loci (QTL) have been identified for multiple types of FHB resistance in wheat with different magnitudes of effects [14–17]. Major and stable QTL often have large effects in multiple environments and are more valuable for practical breeding than minor QTL. However, major and stable QTL are rare for FHB resistance. *Fhb1*, identified from Chinese wheat Wangshuibai and Sumai 3 and located on chromosome 3BS, is the best characterized FHB resistance locus with major effect and stable resistance. *Fhb1* was reported as a pore-forming toxin-like gene (*PFT*) QTL [18]. However, recent studies revealed an histidine-rich calcium-binding protein (*His*) was responsible for the *Fhb1* resistance [19, 20]. A comprehensive discussion on the two studies has been performed, and the nature of *Fhb1* resistance remains unclear [21]. In addition, *Fhb1* has shown linkage with poor agronomic traits and single resistance gene has been proven to be a major limitation for FHB resistance breeding as it may not provide sufficient protection under severe FHB epidemics [15, 22, 23]. Pyramiding *Fhb1* and other major FHB resistance QTL into elite cultivars using MAS could be crucial for breeding new wheat varieties with better resistance to FHB [4, 24]. Several cultivars such as Yangmai158, Yangmai11 Yangmai12, Yangmai16, and Yangmai23 in the Middle and Lower Yangtze River Valleys with moderate resistance to FHB have been approved to be released and become main cultivars [25]. Most of Yangmai-series cultivars don’t carry the *Fhb1* locus [26], indicating that other FHB resistance genes may be present in these cultivars and can be more easily applied to breeding. Therefore, discovering more FHB-resistant germplasms and new FHB-resistant loci is essential for breeding wheat varieties with better FHB resistance.

Genome-wide association studies (GWAS), based on linkage disequilibrium (LD) has been widely used to discover various quantitative traits associated nucleotide polymorphisms in plants. For example, using a panel of 192 bread wheat cultivars from southwest China, 57, 27, 30, and 34 single nucleotide polymorphism (SNP) were identified for associations with plant height (PH), grain protein content (GPC), thousand kernel weight (TKW) and sodium dodecyl sulfate (SDS) content, respectively [27]. One hundred-twenty consistent loci were detected using SNP-GWAS and Haplotype-GWAS, and 78 were potentially new [28]. The recently released reference genome sequence of Chinese Spring [29] provides an elit platform for detecting genes significantly associated with linked markers with known physical positions in the genome and promoting the molecular breeding process [30]. In this study, we report a GWAS analysis of FHB resistance using a set of 171 common wheat cultivars with 90 K SNP genotyping and 2 year’s phenotyping data. The aims of this study were to identify stable loci for FHB resistance using GWAS and better understand the genetic basis of FHB resistance in natural population.

## Results

### Phenotypic variation

**Fig. 1:**
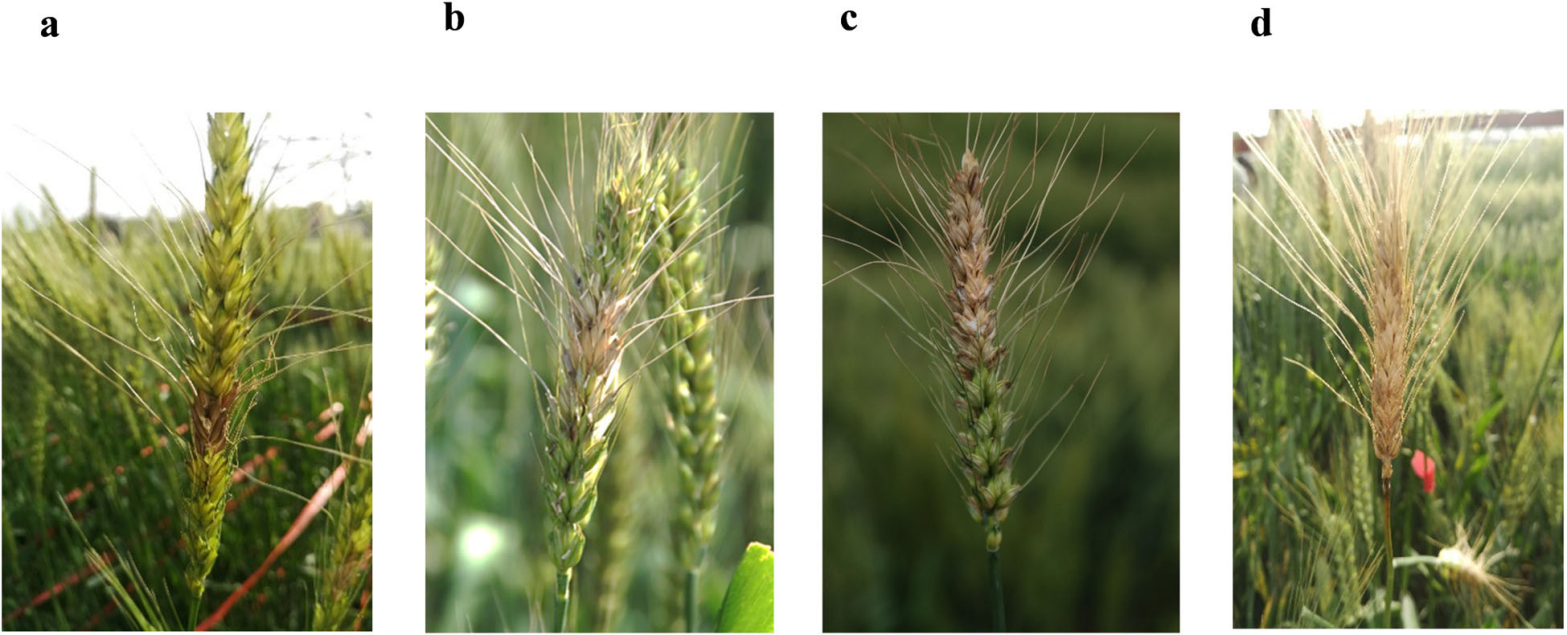
The phenotypic response of the wheat spikelets classified into the four classes based on FHB severity. **a** resistant, **b** moderately resistant, **c** moderately susceptible and **d** susceptible plants

**Fig. 2:**
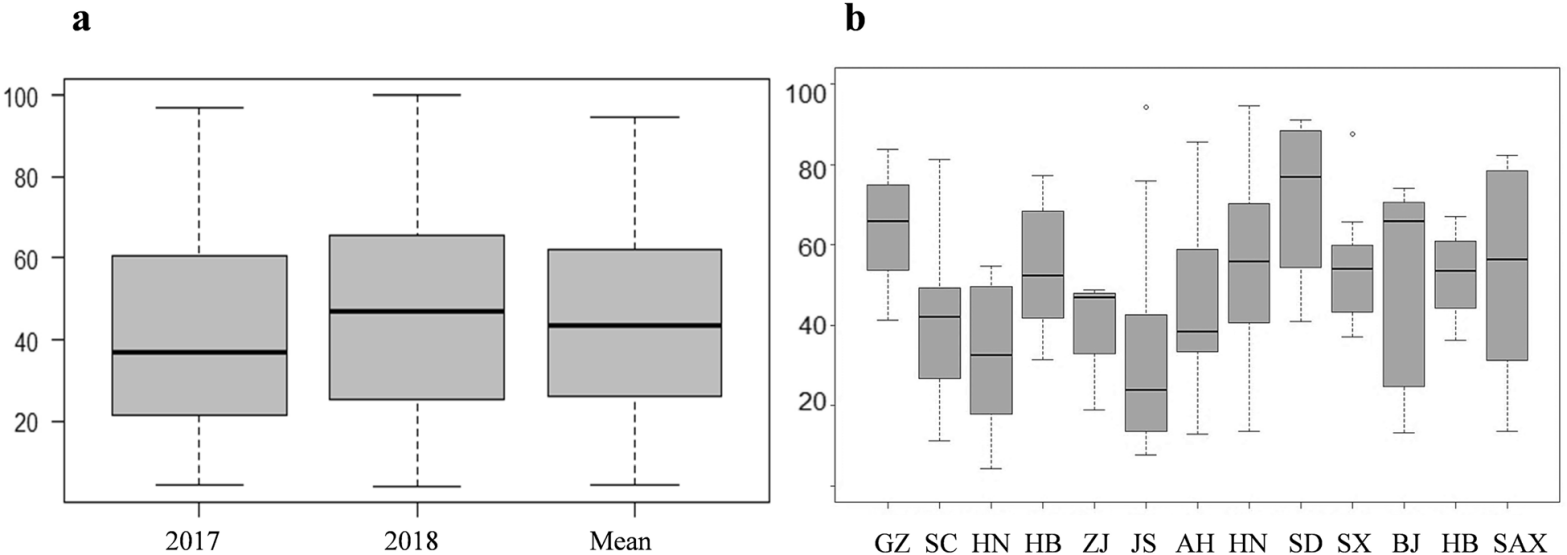
Boxplot of FHB severities. **a** FHB severities of natural population in 2017, 2018 and the mean of the two seasons, y-coordinate indicates the percentage of symptomatic spikelets. **b** FHB severities of wheat cultivars from nine major Chinese provinces, x-coordinate indicates the name of provinces, Guizhou(GZ), Sichuan(SC), Hunan(HN), Hubei(HB), Zhejiang(ZJ), Jiangsu(JS), Anhui(AH), Henan(HN), Shandong(SD), Shanxi(SX), Beijing(BJ), Hebei(HB) and Shaanxi(SAX) in turn from left to right; y-coordinate indicates the percentage of symptomatic spikelets

Continuous variation for percentage of symptomatic spikelets (PSS) was observed at the GWAS panel in both 2017 and 2018 growing seasons, from highly resistant (PSS < 25%) to highly susceptible (PSS > 75%)(Fig. 1). The disease symptom was more severe in 2018 growing season (Fig. 2a). Wheat cultivars from different provinces of China exhibited different levels of resistance to FHB(Fig. 2b). Cultivars from Hunan and Jiangsu provinces exhibited consistently highly resistant to FHB in two seasons, whereas cultivars from Shandong province showed the highest susceptibility.

#### Population structure analysis

**Fig. 3:**
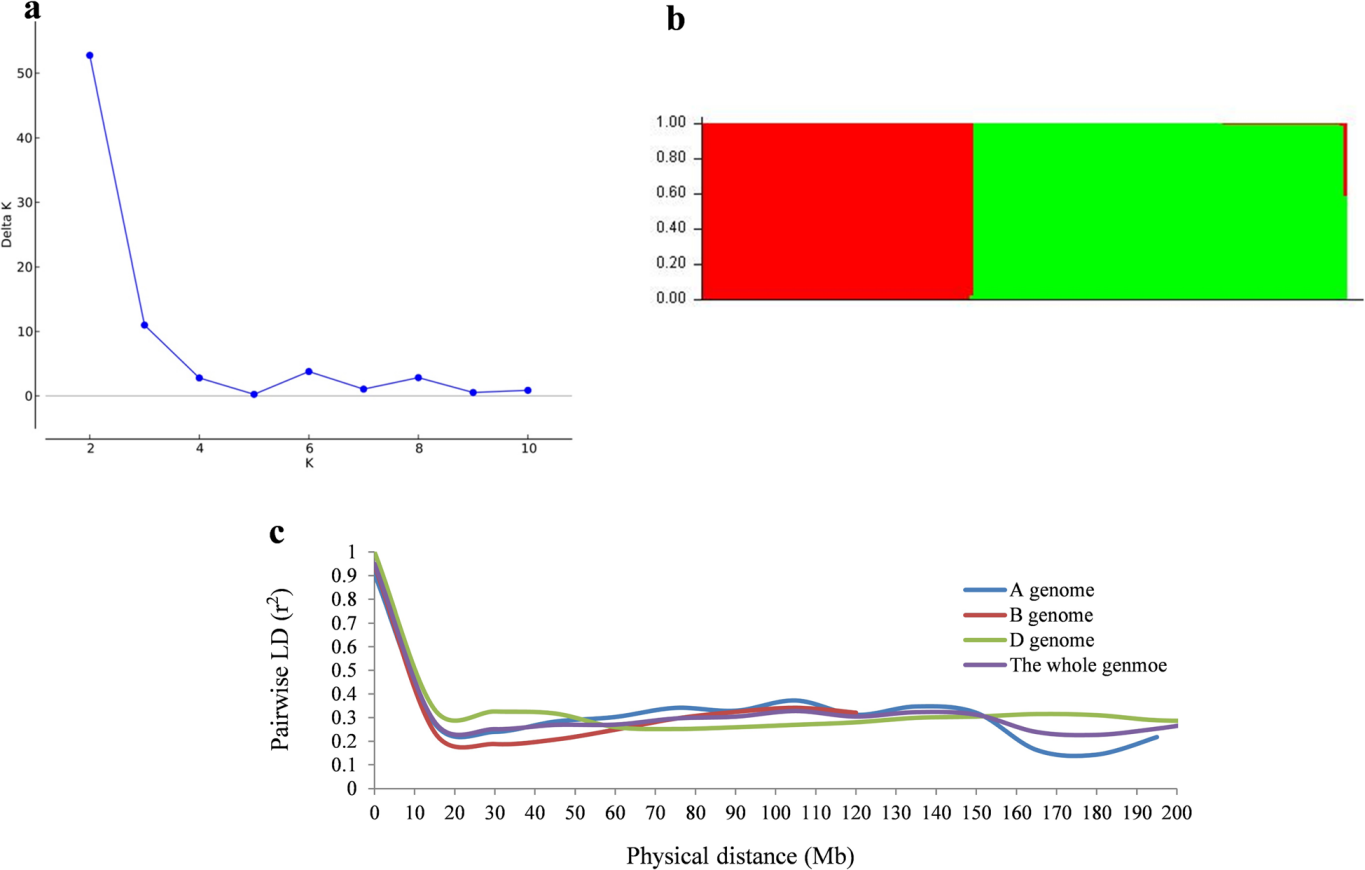
Population structure of 171 wheat cultivars based on 1676 polymorphic SNP markers with whole-genome coverage. **a** Number of subpopulations estimated by ∆K at a range of K-values, and (**b)** Genetic structure produced by Structure V2.3.2. **c** Estimated LD decay for 171 wheat accessions based on filtered markers from the Wheat 90 K array

To estimate the sub-populations of the 171 wheat cultivars, population structure analysis was performed using 1676 polymorphic SNP markers distributing on 21 wheat chromosomes with *r*^*2*^ values > 0.2. The results indicated that the cultivars could be separated into two sub-populations (K = 2) (Fig. 3a, b). Subgroup 1 consists of 99 cultivars, mainly comprising varieties from Anhui, Jiangsu, Henan, Shaanxi and Hunan; subgroup 2 consists 72 cultivars (Additional file 1: Table S1), most of which were from Henan, Jiangsu, Shandong, Shanxi. Wheat cultivars from Anhui and Hunan were all clustered into subgroup 1.

#### Linkage disequilibrium (LD) analysis

The filtered markers from the 90 K SNP genotyping arrays were used to calculated LD decay for the A, B, and D sub-genomes separately as well as the whole genome. 38.9% of all pairs of loci had significant LD (*P* < 0.001) with an average *r*^2^ of 0.281 from 23,556 polymorphic SNPs which distributed at the genome-wide level. The B sub-genome contained the largest number of significant markers (50.0%), followed by A (39.7%) and D (24.0%) sub-genomes. The highest LD decay distance was present in the D sub-genome and the lowest was found in the B sub-genome. The average LD decay distance was ~ 10.5 Mb for the whole genome and 10, 9.5, and 12 Mb for A, B, and D sub-genomes, respectively (Fig. 3c).

### Marker-trait associations

**Fig. 4:**
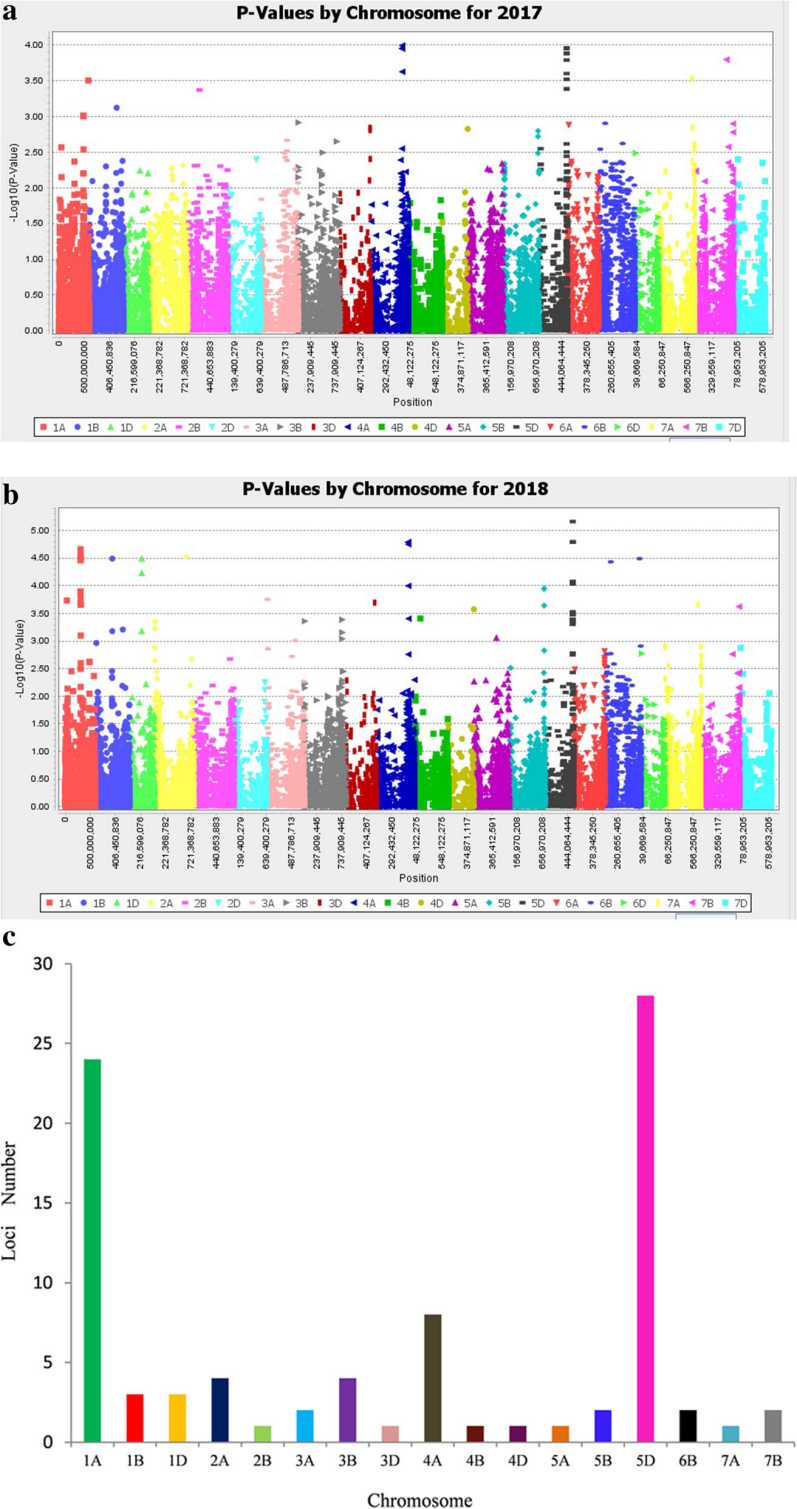
Manhattan plots from genome-wide association scan for FHB severities among 171 wheat accessions in (**a**) 2017 and (**b**) 2018. Dashed horizontal line is the significant threshold level. **c** Numbers of significant FHB associated markers on different chromosomes

Association analysis was conducted using PSS data across 2 years and 23,556 filtered markers. Altogether, 26 loci (88 MTAs, *P* < 10^− 3^) with phenotypic variances explained (*R*^2^) ranging from 6.64–14.18% were identified across all of the chromosomes except for 2D, 6A, 6D and 7D (Fig. 4a, b). Among these, 41, 32 and 15 significant markers were located on the A, D and B sub-genomes, respectively (Fig. 4c; Additional file 1: Table S2). More FHB MTAs were found on chromosomes 1A, 1D, 1B, 2A, 3B, 4A and 5D.

Twenty-eight MTAs located on chromosomes 1B (1), 4A (7), 5D (19) and 7A (1) were consistently identified in both seasons and could be considered as stable QTL (Table 1), SNP *GENE-0293_154* located on 1B explained 6.91–7.18% of phenotypic variances (*R*^2^). Seven and 19 SNPs located on 4AL and 5DL chromosomes explained phenotypic variances (*R*^2^) ranging from 9.36–11.63% and 8.11–14.18%, respectively. The SNP *BobWhite_c22875_239* located on 7A could explain 8.12–8.53% of phenotypic variances (*R*^2^).

**Table. 1:** FHB resistance loci revealed by GWAS in 2 years

Locus	Marker^a^	Chr^b^	Postion (Mb) ^c^	*P* value	R^2^(%)^d^	Allele	Resistant allele
*QFhb-1B.2*	*GENE-0293_154*	1B	549.47	6.20E-04/7.50E-04	6.91–7.18	C/T	C
*QFhb-4A*	*BS00011469_51*	4A	621.85	1.00E-04/2.40E-04	8.37–9.36	C/T	C
	*Excalibur_c22724_85*	4A	622.2	2.00E-05/1.10E-04	9.33–11.63	C/T	T
	*Kukri_c24695_273*	4A	622.2	2.00E-05/1.10E-04	9.33–11.63	A/G	A
	*Kukri_c1073_91*	4A	622.24	2.00E-05/1.00E-04	9.65–11.53	C/T	T
	*Excalibur_c687_961*	4A	622.24	2.00E-05/1.10E-04	9.33–11.63	A/G	A
	*Excalibur_c687_907*	4A	622.24	2.00E-05/1.10E-04	9.33–11.63	C/T	T
	*Excalibur_c687_886*	4A	622.24	2.00E-05/1.10E-04	9.33–11.63	A/G	G
*QFhb-5D*	*BobWhite_c13030_406*	5D	546.09	2.00E-05/1.30E-04	9.37–11.65	A/G	G
	*BS00079676_51*	5D	546.65	2.00E-05/1.10E-04	9.33–11.63	A/G	A
	*RAC875_c13169_459*	5D	546.65	2.00E-05/1.10E-04	9.33–11.63	C/T	C
	*D_GA8KES401AL4GG_122*	5D	546.65	2.00E-05/1.10E-04	9.33–11.63	C/T	C
	*wsnp_JD_c4438_5568170*	5D	546.69	8.00E-05/1.60E-04	10.76–11.72	A/G/R	A
	*wsnp_JD_c4438_5567972*	5D	546.69	2.00E-05/1.10E-04	9.33–11.63	A/G	A
	*wsnp_JD_c4438_5567834*	5D	546.69	9.00E-05/4.10E-04	9.33–11.63	C/T/Y	C
	*BobWhite_c4438_162*	5D	546.69	7.00E-06/2.50E-04	8.89–14.18	A/G	G
	*IACX10520*	5D	546.69	2.00E-05/1.10E-04	9.33–11.63	C/T	T
	*BS00088587_51*	5D	546.69	2.00E-05/1.10E-04	9.33–11.63	G/T	G
	*D_GDS7LZN01CBWNE_99*	5D	546.7	2.00E-05/1.10E-04	9.33–11.63	C/T	T
	*Kukri_c5528_603*	5D	546.7	2.00E-05/1.10E-04	9.33–11.63	C/T	C
	*Excalibur_c42190_383*	5D	546.91	4.10E-04/4.40-E04	9.55–9.57	A/G/R	A
	*Excalibur_c28592_377*	5D	546.91	9.00E-05/3.00E-04	8.11–9.47	C/T	T
	*Excalibur_c28592_173*	5D	546.91	4.10E-04/4.40-E04	9.55–9.57	C/T/Y	T
	*Excalibur_c14043_548*	5D	546.91	9.00E-05/3.00E-04	8.11–9.47	C/T	C
	*CAP8_c145_89*	5D	547.27	2.00E-05/1.10E-04	9.33–11.63	C/T	T
	*wsnp_CAP11_c209_198467*	5D	547.27	2.00E-05/1.30E-04	9.37–11.65	A/G	A
	*BS00011794_51*	5D	547.27	2.00E-05/1.30E-04	9.37–11.65	C/T	T
*QFhb-7A*	*BobWhite_c22875_239*	7A	661.3	2.20E-04/3.00E-04	8.12–8.53	C/T	C

^a^ Markers were detected at the threshold -log10 (*P*) = 3.0

^b^ Chr, Chromosome

^c^ Physical positions of SNP markers based on wheat genome sequences from the International Wheat Genome Sequencing Consortium (IWGSC, http://www.wheatgenome.org/)

^d^ Percentage of phenotypic variance explained by the MTA

**Fig. 5:**
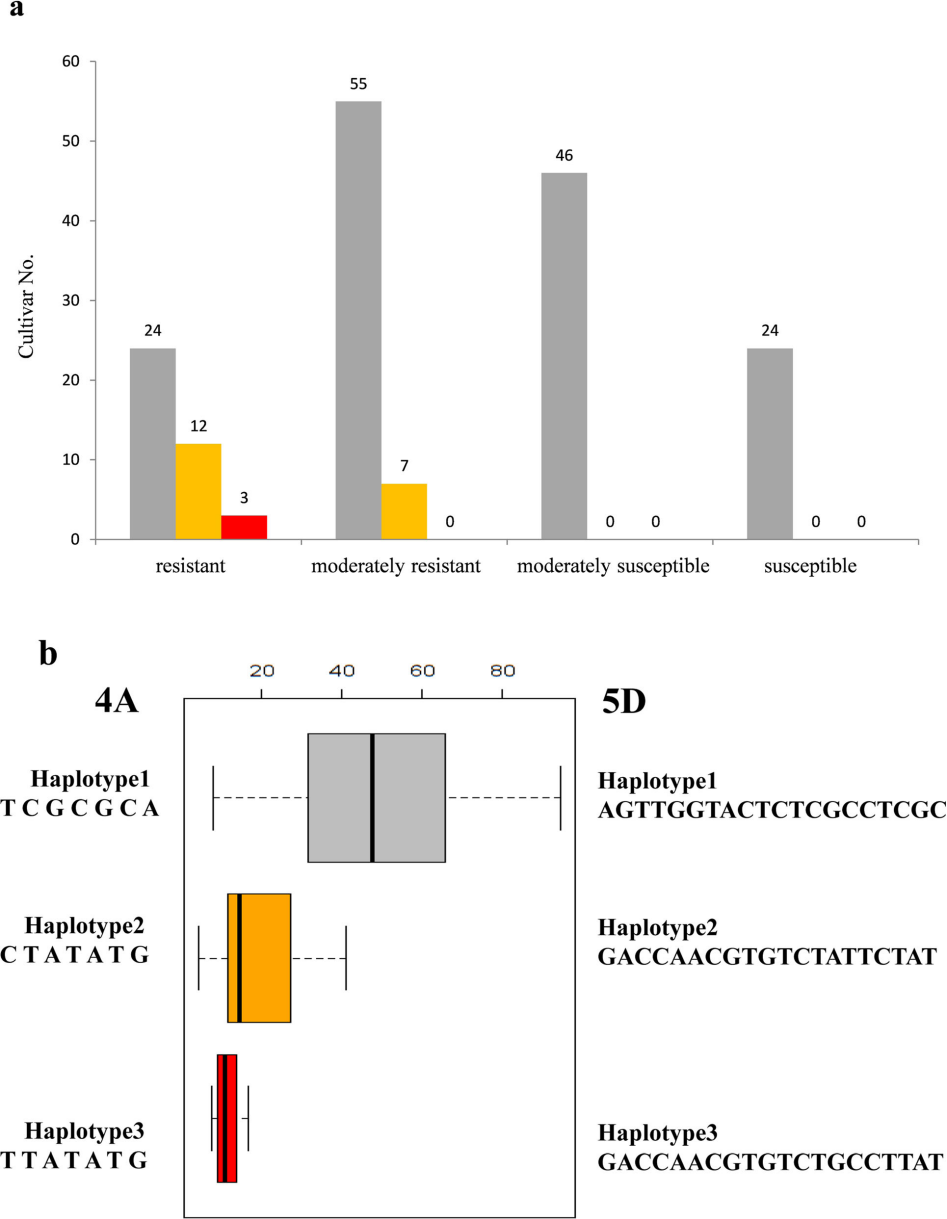
Haplotype analysis results. **a** Frequency distributions of the mean FHB severities for 171 cultivars with different haplotypes on chromosomes 4A and 5D. Gray, orange and red represent haplotype 1, haplotype 2 and haplotype 3, respectively. The x-axis exhibits 1–4 scores based on FHB severity (resistant, 0 < PSS ≤25%; moderately resistant, 25% < PSS ≤50%; moderately susceptible, 50% < PSS ≤75% and susceptible, 75% < PSS ≤ 100%). The y-axis represents the number of cultivars (also numbered on the bar) showing the FHB severity in different haplotypes. **b** Haplotype analysis of significant SNPs on chromosomes 4A and 5D. Solid bar plot displays average FHB severity of each haplotype. Gray, orange and red represent haplotype 1, haplotype 2 and haplotype 3, respectively. Left: Haplotypes of the significant SNPs based on 4A among wheat lines; right: Haplotypes of the significant SNPs based on 5D among wheat lines

Due to the high level of LD in wheat, the SNP clusters identified on chromosomes 4AL (*QFhb-4AL*) from 621.85 Mb to 622.24 Mb and 5DL (*QFhb-5DL*) from 546.09 Mb to 547.27 Mb most likely represented chromosome regions containing significant FHB associated loci, respectively. Haplotype analyses of the associated markers revealed three haplotype groups (Fig. 5a), Haplotype 1 consisted of 149 cultivars with an average PSS of 48.92% over 2 years, in which 24 were resistant, 55 were moderately resistant, and 70 were susceptible. Haplotype 2 consisted of 19 cultivars with an average PSS of 19.94% over 2 years, and 12 of them were resistant and 7 were moderately resistant. Haplotype 3 comprised three resistant cultivars with an average PSS of 11.52%. The results indicated that other resistant genes also existed in the cultivars of Haplotype 1 (Table 2**,** Additional file 1: Table S3). Interestingly, each haplotype contains wheat cultivars with same associated SNPs on both *QFhb-4AL* and *QFhb-5DL* simultaneously **(**Fig. 5b).

**Table. 2:** Descriptive statistics of the three haplotypes for FHB severities

Haplotype^a^	No.^b^	Minimum (%)	Maximum (%)	Mean ^c^ (%)	Standard deviation	Variance
Haplotype1	149	7.71	94.73	48.92A	22.29	496.69
Haplotype2	19	4.29	41.25	19.94B	11.36	129.06
Haplotype3	3	7.3	16.58	11.52B	4.7	22.09

^a^ Three haplotype groups revealed through haplotype analyses of the associated markers

^b^ No., Number of cultivars

^c^ indicates extremely significant differences at 0.01 significance level among parents and controls(*P* < 0.01)

## Discussion

### FHB resistance loci identified by GWAS

QTL for *Fusarium* head blight resistance have been extensively reported using different mapping populations and mapping platforms. From more than 250 documented QTL conferring FHB resistance, only *Fhb1*-*Fhb7* haved been proven to be major effects QTLs. *Qfhs.nau-6B* (*Fhb2*), *Qfhi.nau-4B* (*Fhb4*), and *Qfhi.nau-5A* (*Fhb5*) were fine mapped in the 2.2 cM, 0.14 cM, and 0.09 cM interval [16]. *Fhb1* has been cloned recently [18–20]. In current study, four loci (28 MTAs) were identified on chromosomes 1B, 4A, 5D and 7A in two seasons. In comparison to the SNP *GENE-0293_154* on chromosome 1B identified for type II FHB resistance in this study, a minor QTL for type II resistance was found in a similar physical position from Chinese wheat landrace Huangfangzhu [31]. Two loci located on 4AL and 5DL chromosomes at physical intervals of 0.39 Mb and 1.18 Mb, respectively, were related to type II resistance with variation (*R*^2^) of 9.36–11.63% and 8.11–14.18%, respectively. The SNP *BobWhite_c4438_162* itself on 5DL could explain 8.89–14.18% variation.

QTL for FHB resistance on chromosome 4AL have been reported from European wheat cultivars. Holzapfel et al. [32] identified two FHB resistance QTL on chromosome 4AL from a French cultivar (Apache) linked with *XP7452–646* and a German cultivar (Pirat) linked with *XP7553–254.AR.* Another QTL, *QFhs.fal-4AL*, has been mapped on 4AL at a physical position 357.2 Mb from a Swiss winter wheat cultivar (Arina) [33]*.* FHB QTL identified on 4AL at a physical position from 621.85 Mb to 622.24 Mb in the current study is different from the reported ones and should be new FHB resistance loci. A type II resistance QTL from FHB-resistant wheat cultivar Chokwang (Korea) was mapped on 5DL and linked to the SSR marker *Xbarc239* [34, 35] with a physical position of 420.96 Mb. Jia et al. [36] reported a QTL on chromosome 5D linked with *Xgwm358* with a physical position of 120.61 Mb. Since no QTL for Type II resistance has ever been reported on chromosome 5DL at the physical interval of 546.09 Mb to 547.27 Mb, *QFhb-5DL* is likely to be a new FHB resistance locus. SNPs *wsnp_JD_c4438_5568170* and *wsnp_CAP11_c209_198467* in this QTL region of 5DL were reported to be closely linked to a soil-borne wheat mosaic virus (SBWMV) resistance gene *Sbwm1* [37]. The SNPs have been developed into breeder-friendly Kompetitive Allele-Specific Polymerase chain reaction (KASP) markers for effectively distinguish resistant and susceptible alleles of *Sbwm1* in a diverse wheat panel in breeding programs. It would be interesting to verify whether these KASP markers can be used in marker-assisted selection of FHB resistance in wheat breeding. Furthermore, *BobWhite_c22875_239* was found associated with type II resistance on chromosome 7AL at 661.3 Mb that is about the same proximal region of a reported QTL *QFhb.nau-7A* from Wangshuibai [38, 39] (Additional file 1: Table S4).

### *QFhb-4AL* and *QFhb-5DL* are located on syntenic genomic regions

**Fig. 6:**
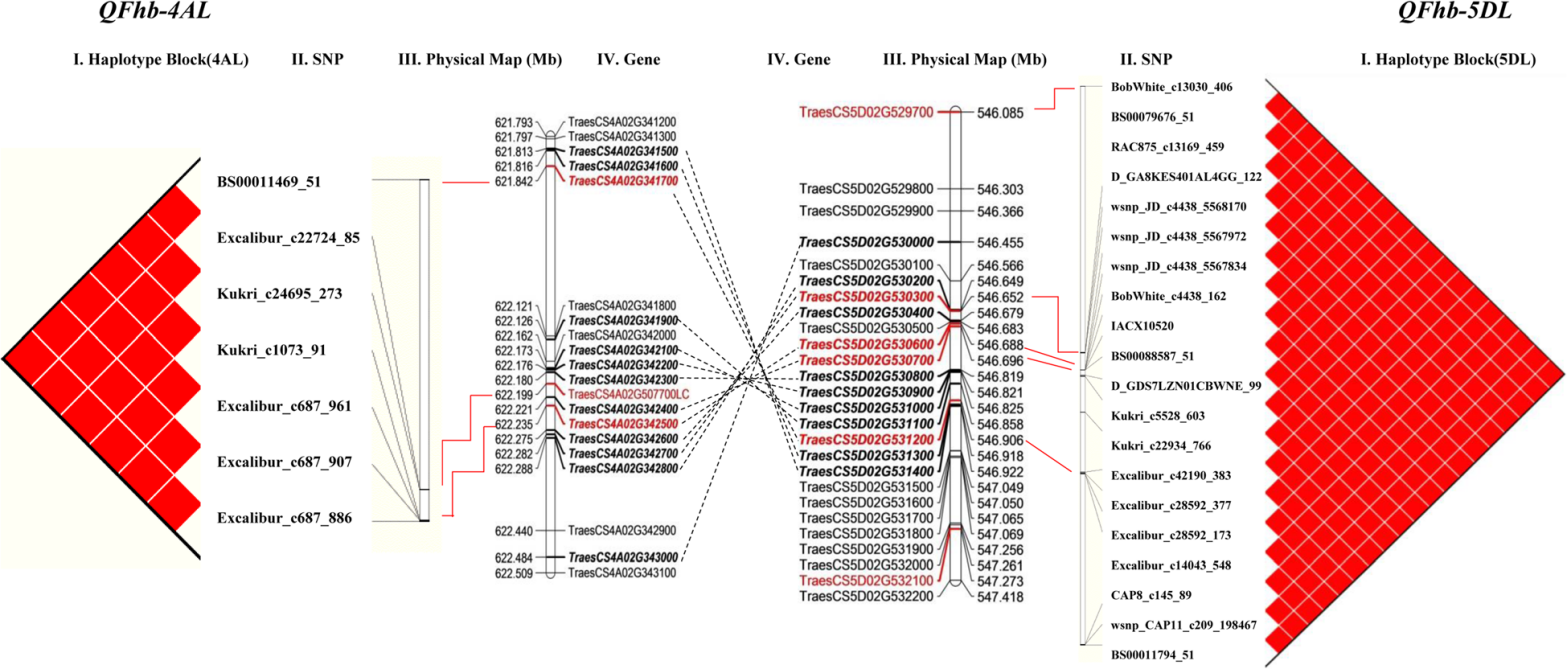
Gene annotation of SNPs identified on chromosome 4AL (**a**) and 5DL (**b**) for FHB resistance. The far left and right image is a visualization of linkage disequilibrium (red is a D’ value of 100%) (I). Names of the markers (II) and physical position (III) were observed in the region of interest. Most significant marker is highlighted in red. The middle image is a physical map of candidate genes on 4AL and 5DL chromosome segments spanning from 621.793 to 622.509 Mb and 546.085 to 547.418 Mb, respectively (IV). The physical position is based on IWGSC 2018. Dotted lines indicate a linear relationship between the significantly associated regions on 4AL and 5DL, respectively

We detected two loci significantly associated with FHB resistance on 4AL and 5DL at a physical intervals of 0.39 Mb and 1.18 Mb, respectively. LD of markers and FHB severity analysis indicated that each haplotype contains wheat cultivars with associated SNP on both *QFhb-4AL* and *QFhb-5DL* simultaneously. Gene annotations of the genomic intervals revealed homologous gene pairs between 4AL and 5DL. Highly collinearity in gene order and content were observed for the two FHB resistant QTL regions, even through large fragment insertions/deletions were also presented (Additional file 1: Table S5**;** Fig. 6).

Wheat has experienced structural evolution involving chromosome translocation of 4A, 5A, and 7B. The 4AL/5AL translocation taken place at the diploid level and existed both in *T. monococcum* and *T. aestivum,* followed by a 4AL/7BS translocation, a pericentric inversion (4AS;4AL) and a paracentric inversion (4AL;4AL) that occurred in the tetraploid progenitor of hexaploid wheat [40]. Recently, Dvorak et al. [41] reassessed the evolution of wheat chromosomes 4A, 5A and 7B after sequence comparison of wild emmer wheat and *Aegilops tauschii*. They found that the 596.20–631.84 Mb genomic region of 4A pseudomolecule was derived from ancestral 5AL with nested inversion and is corresponding to the end of the *Ae*. *tauschii* arm 5DL. The two FHB associated loci on 4AL (621.81–622.49 Mb) and 5DL (546.45–546.92 Mb) are located on the syntenic block with sequence inversion **(**Fig. 6), providing further information of this structure rearrangement containing important genes for agronomic trait.

The hypothetical proteins were predicted for the 4AL and 5DL syntenic blocks **(**Table 3**)**. Two kinase proteins, homologous to PTI1-like tyrosine-protein kinase 1 and Putative receptor protein kinase ZmPK1, proved to be associated with plant disease resistance were annotated in the corresponding genomic regions (Additional file 1: Tables S4 and S5). Protein kinases (PKs) are important for transmembrane signaling that regulates plant development and adaptation to diverse environmental conditions [42]. Several kinase proteins have been reported related to plant innate immunity. For example, the combination of a kinase and a putative START lipid-binding domain is necessary to confer wheat rust resistance of *Yr36* [43]. Wheat stripe rust resistance gene *Yr15* (*WTK1*) [44] and barley (*Hordeum vulgare* L.) stem rust (*P. graminis* f. sp. *tritici*) resistance gene *Rpg1* [45] contain a structure with tandem kinase domains. A maize wall-associated kinase protein (*ZmWAK*) was reported to confer quantitative resistance to maize head smut [46] and the PTI1-like kinase (*ZmPti1*a) was known to play an important role in the signaling pathway that facilitates pollen performance and male fitness [47].

**Table. 3:** Candidate genes for SNPs significantly associated with FHB resistance

Marker^a^	Chr^b^	Position^c^ (cM)	Adjacent *T. aestivum* gene^d^	Predicted function^e^	Identity (%)	Orthologous gene
*GENE-0293_154*	1B	549.47	–	–	–	
*BS00011469_51*	4A	621.85	*TraesCS4A02G341700*	PTI1-like tyrosine-protein kinase	100	*LOC109767070*
*Excalibur_c22724_85 Kukri_c24695_273 Kukri_c1073_91*	4A	622.20	*TraesCS4A02G507700LC*	protein FAR1-RELATED SEQUENCE 6-like	96.62	*LOC109763821*
*Excalibur_c687_961 Excalibur_c687_907 Excalibur_c687_886*	4A	622.24	*TraesCS4A02G342500*	putative receptor protein kinase ZmPK1	99.25	*TRIUR3_00921*
*BobWhite_c13030_406*	5D	546.09	*TraesCS5D02G529700*	uncharacterized protein	100	*LOC109743624*
*BS00079676_51 RAC875_c13169_459 D_GA8KES401AL4GG_122*	5D	546.65	*TraesCS5D01G530300*	putative RNA-binding protein Luc7-like 2	100	*LOC109763817*
*wsnp_JD_c4438_5568170 wsnp_JD_c4438_5567972 wsnp_JD_c4438_5567834 BobWhite_c4438_162 IACX10520 BS00088587_51*	5D	546.69	*TraesCS5D02G530600*	putative receptor protein kinase ZmPK1	100	*LOC109763820*
*D_GDS7LZN01CBWNE_99 Kukri_c5528_603*	5D	546.70	*TraesCS5D02G530700*	probable ion channel CASTOR	100	*LOC109763818*
*Excalibur_c42190_383 Excalibur_c28592_377 Excalibur_c28592_173 Excalibur_c14043_548*	5D	546.91	*TraesCS5D02G531200*	PTI1-like tyrosine-protein kinase	100	*LOC109767070*
*CAP8_c145_89 wsnp_CAP11_c209_198467 BS00011794_51*	5D	547.30	*TraesCS5D02G532100*	uncharacterized protein	99	*LOC109767042*
*BobWhite_c22875_239*	7A	661.30	*TraesCS7A02G464900*	probable polyamine transporter At3g13620	98.73	*LOC109739139*

^a^ Markers were detected at the threshold -log10 (*P*) = 3.0

^b^ Chr, Chromosome

^c^ Physical positions of SNP markers based on wheat genome sequences from the International Wheat Genome Sequencing Consortium (IWGSC, http://www.wheatgenome.org/)

^d^ *T. aestivum* gene transcripts and their domains were explored in Ensembl (using the transcript table link)

^e^ The sequences of *T.aestivum* gene were blasted in the NCBI (http://www.ncbi.nlm.nih.gov/), databases to identify putative gene functions

Kinase proteins were also found to be important in *F. graminearum*. A MAP kinase gene (*MGV1*) in *F. graminearum* was required for much more developmental processes linked to sexual reproduction, plant infection, and cell wall integrity [48]. The glycogen synthase kinase gene orthologous to mammalian *GSK3* was an significant virulence factor and *Fgk3* glycogen synthase kinase was also important for growth, pathogenesis, conidiogenesis, DON production and stress responses in *F. graminearum* [49]. Taken the potential importance of kinase proteins in FHB resistance synthetic loci identified on 4AL and 5DL, the wheat homologs of PTI1-like tyrosine-protein kinase 1 and putative receptor protein kinase ZmPK1 might be considered as candidates of FHB resistance and need further characterization.

## Conclusions

In the present study, we identified 26 FHB resistance loci using the wheat 90 K SNP assay, and four stable loci were detected in both seasons. Two new FHB resistance loci on 4AL and 5DL were found to be located on syntenic genomic regions, indicating that these regions contain important genes valuable for future research and breeding application. The SNP markers significantly associated with the FHB resistance could be used to develop diagnostic markers for marker associated selection of FHB resistance breeding.

## Methods

### Plant materials

An association panel comprising 171 wheat cultivars was used for SNP genotyping and 2 years FHB resistance phenotyping. Among them, three cultivars were derived from Italy, Mexico and Japan, and the other 168 cultivars were collected from 8 provinces at winter wheat region in Northern China and 9 provinces from Southern China (Additional file 1: Table S1). All wheat accessions are collected under permission from the National Genebank of China, Chinese Academy of Agricultural Sciences and Jiangsu Academy of Agricultural Sciences. The population was planted at Wanfu Experimental Station, Institute of Agricultural Sciences of the Lixiahe, Yangzhou, Jiangsu Province, China (altitude 8 m, latitude 32.24°N, annual rainfall about 1000 mm, growing season from early November to the next May) for 5 years, and flowering date were recorded every year. The 171 wheat cultivars displayed a difference of less than 4.0 days on average in flowering date between the earliest cultivar and the latest cultivar.

Field experiments were designed as randomized complete blocks with two replicates per year. The cultivars in each replication were sown in two rows of 133 × 25 cm with 40 seeds per row. The field trials were in accord with local practices management.

### Phenotyping

All cultivars were inoculated in growing seasons 2016–2017 (abbr. as 2017) and 2017–2018 (abbr. as 2018) with four *F. graminearum* strains (F4, F15, F34, and F0609), friendly provided by Prof. Huaigu Chen from Jiangsu Academy of Agricultural Sciences, Nanjing, China. Ten spikes per row were inoculated at the late-heading stage by injecting 10 μL of macroconidial suspension (1.0 × 10^5^ conidia/ml) into a single floret in the middle of each spike based on the flowering time.

The disease nursery was mist-irrigated for 5 min every 30 min from 7:00 am to 6:00 pm each day to ensure the inoculated spikes fully infected under high humid conditions [50]. The number of infected spikelets and the total number of spikelets of every tagged spike were recorded 25 days after inoculation. The average percentage of symptomatic spikelets (PSS) was calculated as the measure of FHB severity. All tested accessions were classified into four classes based on FHB severity, resistant (0 < PSS ≤25%), moderately resistant (25% < PSS ≤50%), moderately susceptible (50% < PSS ≤75%) and susceptible (75% < PSS ≤ 100%) [51].

### Genotyping and SNP calling

Genomic DNA was extracted from fresh leaves of field grown non-infected plants at seedling stage using the CTAB method [52]. The association mapping population was genotyped from the wheat Illumina 90 K iSelect array with 81,587 SNPs (Wang et al. 2014) at the Biotechnology Center, Department of Plant Sciences, University of California, USA, using the Illumina SNP genotyping platform and BeadArray Microbead Chip [53]. To avoid spurious marker-trait associations (MTAs), SNP markers with minor allele frequencies (MAF) < 0.05 and missing data > 10% were excluded from subsequent analyses. The physical positions of SNP markers were obtained from Chinese Spring reference genome sequences at the International Wheat Genome Sequencing Consortium website (IWGSC, http://www.wheatgenome.org/).

### Population structure analysis and linkage disequilibrium

Population structure was estimated using Structure 2.3.4 with 1676 polymorphic SNP markers distributing on all 21 wheat chromosomes with *r*^*2*^ < 0.2, based on the Bayesian cluster analysis [54]. Six runs of Structure were performed with a K between 1 and 11, using the admixture model with 100,000 replicates each for burn-in and MCMC. The optimal K-value was determined using the ∆K method [55].Linkage disequilibrium (LD) among markers was computed by the full matrix and sliding window options in Tassel v5.0 with the filtered SNP markers. The pairwise LD between the markers was calculated using squared allele frequency correlations *r*^*2*^, according to Liu et al. [56].

### GWAS for FHB resistance

Associations between genotypic and phenotypic data were analyzed using the kinship matrix in a Mixed Linear Model (MLM) by Tassel v5.0 to control background variation and eliminate spurious MTAs. The kinship matrix (K matrix) was considered as a random effect factor and the subpopulation data (Q matrix) was considered as a fixed-effect factor in the MLM analysis [57]. The calculation of K matrix and Q matrix was performed using the software Tassel v5.0 and the program Structure v2.3.4. The *R*^*2*^ showing the variation explained by the SNP were recorded [58]. SNPs with an adjusted -log_10_ (*P*-value) ≥3.0 were regarded as significant associated with FHB resistance. Significant SNP markers within one LD on the same chromosome were considered to represent one locus. Haplotype analyses of the significant SNPs were performed with Haploview v.4.2 [59].

### Identification of candidate genes

To identify the candidate genes linked to significant SNPs, the physical positions of the markers preceded by the chromosome name were taken to Ensembl (https://urgi.versailles.inra.fr/gb2/gbrowse/wheat_survey_sequence_annotation), and the genes in the same genetic positions were considered. The intervals were then explored for predicted genes and annotations. For genes that are unavailable from the IWGSC annotations, we evaluated orthologous genes (proteins) in related species with reported predicted functions using the comparative genomics tool in Ensembl. When the genes had less than 70% similar ortholog in the annotated genomes of related species in Ensembl, the sequence of the *T. aestivum* gene was taken to search highly similar sequences using NCBI and basic local alignment search tool (BLAST) (http://blast.ncbi.nlm.nih.gov/Blast.cgi).

## Supplementary information

**Additional file 1: Table S1** 171 wheat accessions used in the genome-wide association study (GWAS) for FHB severities and their origins, **Table S2** Marker-trait associations (MTAs) for FHB resistance in 171 wheat accessions identified by the Tassel v5.0, **Table S3** Cultivars belonging to different haplotype and their FHB severities, **Table S4** Physical positions of reported FHB resistance QTL related to the current study, **Table S5** The associated regions with FHB resistance with the same function exists in the corresponding sections of 4A and 5D.

## Data Availability

The phenotypic data of the current study is available in the Additional file 1: Table S1. The data sets supporting the results of this research could be obtained within the article and its additional files. Any other datasets used and/or analyzed are available upon request.

## Abbreviations

DON: Deoxynivalenol
FHB: *Fusarium* head blight
GPC: Grain protein content
GWAS: Genome-wide association study
His: Histidine-rich calcium-binding protein
LD: Linkage disequilibrium
MAF: Minor allele frequencies
MAS: Marker-assisted selection
MLM: Mixed linear model
MTA: Marker-trait association
PFT: Pore-forming toxin-like gene
PH: Plant height
PSS: The percentage of symptomatic spikelets
QTL: Quantitative trait loci
*R*^*2*^: Phenotypic variance explained
SDS: Sodium dodecyl sulfate
SNP: Single nucleotide polymorphism
TKW: Thousand kernel weight
