# Supplementary material for: Genome-wide association mapping revealed syntenic loci QFhb-4AL and QFhb-5DL for Fusarium head blight resistance in common wheat (Triticum aestivum L.)
Source: BMC Plant Biol. 2020 Jan 20;20:29. doi: 10.1186/s12870-019-2177-0 (PMC6971946; doi:10.1186/s12870-019-2177-0)
Supplement: Supplementary file 1 — Additional file 1: Table S1 171 wheat accessions used in the genome-wide association study (GWAS) for FHB severities and their origins, Table S2 Marker-trait associations (MTAs) for FHB resistance in 171 wheat accessions identified by the Tassel v5.0, Table S3 Cultivars belonging to different haplotype and their FHB severities, Table S4 Physical positions of reported FHB resistance QTL related to the current study, Table S5 The associated regions with FHB resistance with the same function exists in the corresponding sections of 4A and 5D. [file 12870_2019_2177_MOESM1_ESM.doc]

**Additional file 1: Table S1 171 wheat accessions used in the genome-wide association study (GWAS) for FHB severities and their origins**

| **Accession number** | **Cultivar name** | **Origin** | **Subgroup^a^** | **2017^b^** | **2018^b^** |
| --- | --- | --- | --- | --- | --- |
| 1 | Ai 73 | Anhui | 1 | 22.52 | 15.20 |
| 2 | Annong 0721 | Anhui | 1 | 48.53 | 78.61 |
| 3 | Annong 8455 | Anhui | 1 | 70.00 | 47.87 |
| 4 | Annong 92484 | Anhui | 1 | 44.16 | 66.67 |
| 5 | Wanmai 17 | Anhui | 1 | 12.29 | 54.55 |
| 6 | Wanmai 18 | Anhui | 1 | 58.12 | 60.20 |
| 7 | Wanmai 32 | Anhui | 1 | 23.78 | 44.78 |
| 8 | Wanmai 47 | Anhui | 1 | 95.21 | 76.00 |
| 9 | Wanmai 48 | Anhui | 1 | 35.65 | 41.23 |
| 10 | Wanxi 7751 | Anhui | 1 | 50.56 | 37.89 |
| 11 | Sudi 8112 | Anhui | 1 | 30.23 | 25.33 |
| 12 | Wanpin 8056 | Anhui | 1 | 39.91 | 33.63 |
| 13 | Wu 7815-4-1 | Anhui | 1 | 12.95 | 12.73 |
| 14 | Beijing 11 | Beijing | 1 | 15.34 | 24.21 |
| 15 | Beijing 12 | Beijing | 1 | 72.43 | 75.86 |
| 16 | Bobwhite | Unknown | 1 | 29.72 | 37.09 |
| 17 | H35 | Unknown | 1 | 71.50 | 71.89 |
| 18 | K35 | Unknown | 1 | 44.12 | 51.02 |
| 19 | NR98117-20S | Unknown | 1 | 83.00 | 83.25 |
| 20 | N553 | Unknown | 1 | 11.79 | 16.43 |
| 21 | Fazhan 5 | Unknown | 1 | 40.59 | 44.50 |
| 22 | Zang 2726 | Unknown | 1 | 12.56 | 59.19 |
| 23 | Fufan 17 | Fujian | 1 | 22.71 | 29.41 |
| 24 | Fumian 2 | Fujian | 1 | 36.67 | 66.81 |
| 25 | Gan 83(Yuan 45)-5-4-42 | Gansu | 1 | 34.48 | 33.01 |
| 26 | Gan mai 7 | Gansu | 1 | 46.11 | 40.91 |
| 27 | Bimai 6 | Guizhou | 1 | 70.62 | 96.95 |
| 28 | Qianhuan 2 | Guizhou | 1 | 49.46 | 33.04 |
| 29 | Gaocheng 8901 | Hebei | 1 | 86.96 | 47.14 |
| 30 | Shimai 12 | Hebei | 1 | 53.85 | 50.91 |
| 31 | Baiquan 565 | Henan | 1 | 85.29 | 78.11 |
| 32 | Boai 7023 | Henan | 1 | 36.76 | 65.09 |
| 33 | Suinong 3 | Henan | 1 | 97.00 | 70.94 |
| 34 | Wenmai 8 | Henan | 1 | 85.22 | 83.02 |
| 35 | Yubao 11 | Henan | 1 | 31.16 | 50.00 |
| 36 | Zhouyou 102 | Henan | 1 | 52.76 | 69.63 |
| 37 | Yanshi 4 | Henan | 1 | 29.28 | 40.50 |
| 38 | Hechun 12 | Heilongjiang | 1 | 56.99 | 66.34 |
| 39 | E1161 | Hubei | 1 | 30.43 | 67.14 |
| 40 | Emai 9 | Hubei | 1 | 50.51 | 12.42 |
| 41 | Jingzhou 66 | Hubei | 1 | 47.21 | 64.50 |
| 42 | Xiangmai 48 | Hubei | 1 | 55.61 | 81.19 |
| 43 | Xiangmai 5 | Hubei | 1 | 32.84 | 50.71 |
| 44 | Xiang 1437 | Hunan | 1 | 35.24 | 52.58 |
| 45 | Xiang 1479 | Hunan | 1 | 56.76 | 52.49 |
| 46 | Xiang 673-1 | Hunan | 1 | 49.24 | 52.17 |
| 47 | Xiang 1599 | Hunan | 1 | 34.45 | 62.96 |
| 48 | Xiang IR(2) | Hunan | 1 | 21.69 | 23.62 |
| 49 | Xiangnong 7984-26-1 | Hunan | 1 | 65.92 | 42.67 |
| 50 | Xiang 675-2 | Hunan | 1 | 12.97 | 38.58 |
| 51 | Xiangmai 10 | Hunan | 1 | 7.07 | 15.60 |
| 52 | Xiangnong 153-27 | Hunan | 1 | 9.36 | 16.55 |
| 53 | Xiangnong 13744-(19-22) | Hunan | 1 | 4.42 | 4.15 |
| 54 | Xiangnong 4053-16-0-20 | Hunan | 1 | 19.30 | 45.74 |
| 55 | Zhenmai 4 | Jiangsu | 1 | 45.35 | 46.59 |
| 56 | Sumai 2 | Jiangsu | 1 | 11.89 | 26.96 |
| 57 | Xumai 27 | Jiangsu | 1 | 88.52 | 100.00 |
| 58 | Xuzhou 15 | Jiangsu | 1 | 40.63 | 40.37 |
| 59 | Xuzhou 8785 | Jiangsu | 1 | 32.65 | 77.45 |
| 60 | Xuan 7 | Jiangsu | 1 | 67.15 | 84.54 |
| 61 | Yangfumai 2 | Jiangsu | 1 | 7.45 | 34.39 |
| 62 | Yangfumai 4 | Jiangsu | 1 | 21.28 | 36.54 |
| 63 | Yangfumai 5242 | Jiangsu | 1 | 18.48 | 29.25 |
| 64 | Yangmai 10 | Jiangsu | 1 | 18.09 | 19.90 |
| 65 | Yangmai 11 | Jiangsu | 1 | 18.13 | 16.56 |
| 66 | Yangmai 13 | Jiangsu | 1 | 55.33 | 51.28 |
| 67 | Yangmai 14 | Jiangsu | 1 | 10.81 | 7.65 |
| 68 | Yangmai 9 | Jiangsu | 1 | 17.73 | 35.68 |
| 69 | Zhenmai 6 | Jiangsu | 1 | 6.38 | 10.88 |
| 70 | Xuzhou 20 | Jiangsu | 1 | 92.57 | 58.50 |
| 71 | Ningmai 13 | Jiangsu | 1 | 11.87 | 16.97 |
| 72 | Sumai 5 | Jiangsu | 1 | 15.96 | 7.35 |
| 73 | Sumai 6 | Jiangsu | 1 | 16.85 | 16.15 |
| 74 | Yang 85-85 | Jiangsu | 1 | 23.47 | 26.63 |
| 75 | Yanfu 188 | Shandong | 1 | 92.79 | 89.27 |
| 76 | Yannong 19 | Shandong | 1 | 45.11 | 36.88 |
| 77 | Jinmai 33 | Shanxi | 1 | 59.57 | 51.49 |
| 78 | Taiyuan 567 | Shanxi | 1 | 51.39 | 35.65 |
| 79 | Shangluo 76(57)22-0-8-7-2 | Shaanxi | 1 | 87.21 | 77.34 |
| 80 | Bima 1 | Shaanxi | 1 | 66.49 | 89.20 |
| 81 | Bima 4 | Shaanxi | 1 | 66.18 | 97.93 |
| 82 | Shaannong 17 | Shaanxi | 1 | 68.04 | 73.98 |
| 83 | Shaannong 21 | Shaanxi | 1 | 52.86 | 46.19 |
| 84 | Xi'anshixinmai | Shaanxi | 1 | 21.56 | 26.12 |
| 85 | Xinong 2611 | Shaanxi | 1 | 65.38 | 18.89 |
| 86 | Xiaoyan 168 | Shaanxi | 1 | 27.57 | 24.27 |
| 87 | Xiaoyan 54 | Shaanxi | 1 | 11.35 | 15.71 |
| 88 | Xiaoyan 5 | Shaanxi | 1 | 64.73 | 93.53 |
| 89 | Yanda 25 | Shaanxi | 1 | 10.92 | 62.38 |
| 90 | Xiaoyan 96 | Shaanxi | 1 | 87.68 | 38.86 |
| 91 | Chuan 78001 | Sichuan | 1 | 22.82 | 44.61 |
| 92 | Chuan 80-466 | Sichuan | 1 | 28.34 | 58.42 |
| 93 | Chuanmai 37 | Sichuan | 1 | 27.75 | 9.91 |
| 94 | Chuanmai 41 | Sichuan | 1 | 13.47 | 9.00 |
| 95 | Chuanyu 16 | Sichuan | 1 | 13.24 | 29.68 |
| 96 | Chuanyu 21526 | Sichuan | 1 | 44.39 | 43.24 |
| 97 | Chuan 9920 | Sichuan | 1 | 50.00 | 34.40 |
| 98 | Haining 75-39 | Zhejiang | 1 | 28.57 | 69.15 |
| 99 | Xifeng | Japan | 1 | 10.94 | 28.16 |
| 100 | Kechun 5 | Beijing | 2 | 71.43 | 75.69 |
| 101 | Zhongluotiegan | Beijing | 2 | 33.90 | 25.16 |
| 102 | Nongda 116 | Beijing | 2 | 65.85 | 63.90 |
| 103 | Youmangbai 4 | Beijing | 2 | 58.82 | 76.53 |
| 104 | Youmanghong 18 | Beijing | 2 | 75.66 | 57.95 |
| 105 | Zhongyou 16 | Beijing | 2 | 9.44 | 16.83 |
| 106 | Qianhuan 3 | Guizhou | 2 | 82.81 | 49.06 |
| 107 | Henong 822 | Hebei | 2 | 42.86 | 29.76 |
| 108 | Jimai 24 | Hebei | 2 | 45.96 | 63.29 |
| 109 | Huapei 128-8 | Henan | 2 | 72.65 | 45.21 |
| 110 | Huapei 128 baida | Henan | 2 | 41.31 | 41.15 |
| 111 | Kaifeng 10 | Henan | 2 | 94.42 | 27.33 |
| 112 | Yu 30691-1-3 | Henan | 2 | 33.99 | 47.87 |
| 113 | Yu 30691-3-6 | Henan | 2 | 32.26 | 57.14 |
| 114 | Yu 85-2325 | Henan | 2 | 47.87 | 70.10 |
| 115 | Yumai 10 | Henan | 2 | 22.35 | 51.28 |
| 116 | Yumai 13 | Henan | 2 | 56.22 | 55.41 |
| 117 | Yumai 17 | Henan | 2 | 29.86 | 22.40 |
| 118 | Yumai 50 | Henan | 2 | 86.08 | 62.56 |
| 119 | Zheng 87302-0-12-A | Henan | 2 | 36.32 | 24.33 |
| 120 | Zhengmai 004 | Henan | 2 | 95.52 | 93.93 |
| 121 | Zhengzhou 741 | Henan | 2 | 61.88 | 78.70 |
| 122 | Zhengzhou 9285 | Henan | 2 | 11.65 | 15.42 |
| 123 | Jingzhou 1 | Hubei | 2 | 88.33 | 66.18 |
| 124 | Ningai8628 | Jiangsu | 2 | 34.34 | 56.50 |
| 125 | Ningmai 10 | Jiangsu | 2 | 6.88 | 17.24 |
| 126 | Ningmai 11 | Jiangsu | 2 | 9.55 | 5.86 |
| 127 | Ningmai 12 | Jiangsu | 2 | 86.46 | 37.98 |
| 128 | Ningmai 3 | Jiangsu | 2 | 29.67 | 25.23 |
| 129 | Ningmai 6 | Jiangsu | 2 | 30.57 | 27.54 |
| 130 | Ningmai 8 | Jiangsu | 2 | 44.44 | 41.99 |
| 131 | Yangmai 16 | Jiangsu | 2 | 10.40 | 23.53 |
| 132 | Yangmai 19 | Jiangsu | 2 | 7.95 | 13.27 |
| 133 | Yangmai 2 | Jiangsu | 2 | 30.69 | 21.35 |
| 134 | Yangmai 3 | Jiangsu | 2 | 29.79 | 22.95 |
| 135 | Yangmai 6 | Jiangsu | 2 | 6.80 | 20.10 |
| 136 | Zhen 7495 | Jiangsu | 2 | 26.23 | 21.82 |
| 137 | Zhen 7630 | Jiangsu | 2 | 54.59 | 51.64 |
| 138 | Zhenmai 1 | Jiangsu | 2 | 31.22 | 54.17 |
| 139 | Ningmai 9 | Jiangsu | 2 | 24.73 | 32.99 |
| 140 | Ningmai18 | Jiangsu | 2 | 44.44 | 41.99 |
| 141 | Yangmai 18 | Jiangsu | 2 | 10.38 | 10.00 |
| 142 | Yangmai 1 | Jiangsu | 2 | 13.88 | 7.18 |
| 143 | Zhenmai168 | Jiangsu | 2 | 19.27 | 13.89 |
| 144 | Zhenmai9 | Jiangsu | 2 | 6.77 | 9.55 |
| 145 | Zhenmai 5 | Jiangsu | 2 | 5.31 | 16.88 |
| 146 | Jimai 20 | Shandong | 2 | 74.19 | 94.06 |
| 147 | Jinan 14 | Shandong | 2 | 94.68 | 82.35 |
| 148 | Jinan 16 | Shandong | 2 | 90.24 | 63.50 |
| 149 | Jinan 8 | Shandong | 2 | 56.86 | 88.79 |
| 150 | Linmai 2 | Shandong | 2 | 73.08 | 80.93 |
| 151 | Lumai 2 | Shandong | 2 | 96.74 | 85.42 |
| 152 | Lumai 3 | Shandong | 2 | 31.98 | 57.87 |
| 153 | Shannongfu 63 | Shandong | 2 | 52.15 | 56.38 |
| 154 | Jinmai 52 | Shanxi | 2 | 46.35 | 61.62 |
| 155 | Pubing 04-3507 | Shanxi | 2 | 91.62 | 83.51 |
| 156 | Pubing 4302 | Shanxi | 2 | 43.78 | 79.21 |
| 157 | Pubing 4313 | Shanxi | 2 | 25.23 | 49.03 |
| 158 | Shan 253 | Shanxi | 2 | 36.65 | 49.25 |
| 159 | Shan 76(73)33-6 | Shanxi | 2 | 21.84 | 94.61 |
| 160 | Shan 7859 | Shanxi | 2 | 48.07 | 83.42 |
| 161 | Shan 8242-37 | Shanxi | 2 | 36.65 | 68.90 |
| 162 | Shanmai 150 | Shanxi | 2 | 20.49 | 63.33 |
| 163 | Miannong 4 | Sichuan | 2 | 74.50 | 88.21 |
| 164 | Mianyang 15 | Sichuan | 2 | 69.68 | 65.30 |
| 165 | Mianyang 30 | Sichuan | 2 | 46.51 | 63.27 |
| 166 | Mianyang 31 | Sichuan | 2 | 39.34 | 24.51 |
| 167 | Yunmai 27 | Yunnan | 2 | 70.42 | 62.98 |
| 168 | Zhefeng 2 | Zhejiang | 2 | 42.16 | 51.71 |
| 169 | Zhenongda 85B | Zhejiang | 2 | 18.23 | 19.38 |
| 170 | St1472/506 | Italy | 2 | 13.92 | 37.80 |
| 171 | Mexipak 66 | Mexico | 2 | 90.45 | 75.59 |

a Accessions were grouped into subgroups 1, 2 using 1,676 polymorphic SNP markers distributing on 21 wheat chromosomes with *r^2^* values >0.2

b Average FHB severity in 2017 and 2018

**Additional file 2: Table S2** Marker-trait associations (MTAs) for FHB resistance in 171 wheat accessions identified by the Tassel v5.0

| **SNP marker^a^** | **Chromosome** | **Physical Position ^b^ (Mb)** | ***P* value** | ***R^2^* ^c^(%)** | **Environment** | **Resistance allele** |
| --- | --- | --- | --- | --- | --- | --- |
| *RAC875_c24163_501* | 1A | 32.55 | 1.84E-04 | 8.62 | 2018 | C |
| *BS00022156_51* | 1A | 301.2 | 3.37E-05 | 11.86 | 2018 | A |
| *Tdurum_contig43360_1592* | 1A | 301.2 | 3.23E-05 | 10.82 | 2018 | A |
| *Tdurum_contig43360_1381* | 1A | 301.2 | 2.19E-04 | 10.45 | 2018 | C |
| *IAAV4916* | 1A | 301.78 | 2.20E-05 | 11.47 | 2018 | T |
| *BS00031200_51* | 1A | 301.78 | 1.37E-04 | 9.82 | 2018 | T |
| *Ra_c2322_174* | 1A | 301.86 | 1.27E-04 | 9.08 | 2018 | G |
| *Ra_c2322_1067* | 1A | 301.86 | 1.84E-04 | 8.62 | 2018 | T |
| *IACX7695* | 1A | 301.9 | 1.31E-04 | 9.05 | 2018 | A |
| *wsnp_Ex_c1959_3692097* | 1A | 302.63 | 2.86E-05 | 11.07 | 2018 | A |
| *IAAV3552* | 1A | 304.04 | 3.23E-05 | 10.82 | 2018 | A |
| *Kukri_c28532_103* | 1A | 304.04 | 3.48E-05 | 11.83 | 2018 | G |
| *wsnp_Ex_c2178_4086161* | 1A | 305 | 1.53E-04 | 11.08 | 2018 | T |
| *RAC875_c41581_176* | 1A | 305 | 2.20E-05 | 11.47 | 2018 | A |
| *CAP7_c3885_73* | 1A | 305.33 | 3.23E-05 | 10.82 | 2018 | T |
| *Ra_c10580_1629* | 1A | 307.71 | 3.23E-05 | 10.82 | 2018 | C |
| *RAC875_c49760_107* | 1A | 308.21 | 7.94E-04 | 9.27 | 2018 | G |
| *BS00095100_51* | 1A | 308.57 | 3.20E-05 | 10.83 | 2018 | T |
| *Tdurum_contig81558_272* | 1A | 308.81 | 2.19E-04 | 10.45 | 2018 | A |
| *wsnp_Ku_rep_c71909_71634013* | 1A | 308.81 | 2.19E-04 | 10.45 | 2018 | C |
| *Kukri_c60564_136* | 1A | 482.25 | 9.86E-04 | 6.67 | 2017 | T |
| *BS00094925_51* | 1A | 482.97 | 9.86E-04 | 6.67 | 2017 | C |
| *IACX742* | 1A | 483.03 | 9.62E-04 | 7.83 | 2017 | T |
| *IACX882* | 1A | 581.44 | 3.14E-04 | 7.94 | 2017 | C |
| *IACX8300* | 1B | 341.93 | 6.62E-04 | 7.12 | 2018 | T |
| *GENE-0405_237* | 1B | 342.65 | 3.23E-05 | 10.82 | 2018 | T |
| *GENE-0293_154* | 1B | 549.47 | 6.20E-04 | 7.18 | 2018 | C |
| *GENE-0293_154* | 1B | 549.47 | 7.50E-04 | 6.91 | 2017 | C |
| *BS00009866_51* | 1D | 236.44 | 6.62E-04 | 7.12 | 2018 | A |
| *wsnp_RFL_Contig2808_2585736* | 1D | 238.07 | 3.23E-05 | 10.82 | 2018 | G |
| *IACX10924* | 1D | 238.69 | 5.91E-05 | 10.27 | 2018 | T |
| *tplb0032i02_1435* | 2A | 56.77 | 4.48E-04 | 7.56 | 2018 | A |
| *wsnp_Ra_c21104_30458226* | 2A | 56.77 | 4.48E-04 | 7.56 | 2018 | T |
| *Ra_c510_171* | 2A | 59.12 | 5.97E-04 | 7.22 | 2018 | T |
| *BobWhite_c478_1386* | 2A | 635.6 | 3.02E-05 | 10.93 | 2018 | G |
| *Tdurum_contig19413_1144* | 2B | 232.26 | 4.25E-04 | 7.58 | 2017 | T |
| *BS00062963_51* | 3A | 14.76 | 1.75E-04 | 9.26 | 2018 | T |
| *BobWhite_c6265_407* | 3A | 562.29 | 9.76E-04 | 6.65 | 2018 | T |
| *wsnp_Ex_c8386_14128029* | 3B | 71.88 | 4.35E-04 | 7.84 | 2018 | A |
| *Excalibur_c18410_251* | 3B | 752.09 | 4.09E-04 | 7.91 | 2018 | G |
| *Excalibur_c18410_136* | 3B | 752.09 | 6.96E-04 | 7.05 | 2018 | T |
| *Excalibur_rep_c102300_102* | 3B | 752.09 | 9.11E-04 | 6.83 | 2018 | A |
| *Excalibur_rep_c70658_301* | 3D | 564.31 | 2.01E-04 | 8.64 | 2018 | C |
| *BS00011469_51* | 4A | 621.85 | 1.00E-04 | 9.36 | 2018 | C |
| *BS00011469_51* | 4A | 621.85 | 2.40E-04 | 8.37 | 2017 | C |
| *Excalibur_c22724_85* | 4A | 622.2 | 2.00E-05 | 11.63 | 2018 | T |
| *Excalibur_c22724_85* | 4A | 622.2 | 1.10E-04 | 9.33 | 2017 | T |
| *Kukri_c24695_273* | 4A | 622.2 | 2.00E-05 | 11.63 | 2018 | A |
| *Kukri_c24695_273* | 4A | 622.2 | 1.10E-04 | 9.33 | 2017 | A |
| *Kukri_c1073_91* | 4A | 622.24 | 2.00E-05 | 11.53 | 2018 | T |
| *Kukri_c1073_91* | 4A | 622.24 | 1.00E-04 | 9.65 | 2017 | T |
| *Excalibur_c687_961* | 4A | 622.24 | 2.00E-05 | 11.63 | 2018 | A |
| *Excalibur_c687_961* | 4A | 622.24 | 1.10E-04 | 9.33 | 2017 | A |
| *Excalibur_c687_907* | 4A | 622.24 | 2.00E-05 | 11.63 | 2018 | T |
| *Excalibur_c687_907* | 4A | 622.24 | 1.10E-04 | 9.33 | 2017 | T |
| *Excalibur_c687_886* | 4A | 622.24 | 2.00E-05 | 11.63 | 2018 | G |
| *Excalibur_c687_886* | 4A | 622.24 | 1.10E-04 | 9.33 | 2017 | G |
| *Kukri_rep_c106490_1398* | 4A | 626.32 | 3.91E-04 | 7.78 | 2018 | A |
| *Excalibur_c6227_1073* | 4B | 115.39 | 3.89E-04 | 8.08 | 2018 | C |
| *BS00067206_51* | 4D | 507.48 | 2.65E-04 | 8.84 | 2018 | A |
| *RAC875_c15713_943* | 5A | 447.54 | 8.70E-04 | 6.78 | 2018 | A |
| *CAP12_c5949_104* | 5B | 689.85 | 1.13E-04 | 9.57 | 2018 | C |
| *BS00067308_51* | 5B | 690.33 | 2.27E-04 | 8.79 | 2018 | G |
| *Excalibur_c36048_410* | 5D | 542.69 | 4.93E-04 | 7.57 | 2018 | G |
| *Excalibur_rep_c109856_92* | 5D | 542.7 | 4.03E-04 | 7.68 | 2018 | C |
| *Excalibur_c22576_108* | 5D | 542.7 | 4.03E-04 | 7.68 | 2018 | G |
| *D_GB5Y7FA02JRQ1I_101* | 5D | 542.99 | 3.33E-04 | 9.01 | 2018 | A |
| *BobWhite_c8945_134* | 5D | 543.06 | 2.94E-04 | 10.51 | 2018 | C |
| *CAP7_c3364_401* | 5D | 543.06 | 4.21E-04 | 7.83 | 2018 | A |
| *BobWhite_c13030_406* | 5D | 546.09 | 2.00E-05 | 11.65 | 2018 | G |
| *BobWhite_c13030_406* | 5D | 546.09 | 1.30E-04 | 9.37 | 2017 | G |
| *BS00079676_51* | 5D | 546.65 | 2.00E-05 | 11.63 | 2018 | A |
| *BS00079676_51* | 5D | 546.65 | 1.10E-04 | 9.33 | 2017 | A |
| *RAC875_c13169_459* | 5D | 546.65 | 2.00E-05 | 11.63 | 2018 | C |
| *RAC875_c13169_459* | 5D | 546.65 | 1.10E-04 | 9.33 | 2017 | C |
| *D_GA8KES401AL4GG_122* | 5D | 546.65 | 2.00E-05 | 11.63 | 2018 | C |
| *D_GA8KES401AL4GG_122* | 5D | 546.65 | 1.10E-04 | 9.33 | 2017 | C |
| *Kukri_c7786_81* | 5D | 546.69 | 3.30E-04 | 8.44 | 2018 | T |
| *wsnp_JD_c4438_5568170* | 5D | 546.69 | 8.00E-05 | 11.72 | 2018 | A |
| *wsnp_JD_c4438_5568170* | 5D | 546.69 | 1.60E-04 | 10.76 | 2017 | A |
| *wsnp_JD_c4438_5567972* | 5D | 546.69 | 2.00E-05 | 11.63 | 2018 | A |
| *wsnp_JD_c4438_5567972* | 5D | 546.69 | 1.10E-04 | 9.33 | 2017 | A |
| *wsnp_JD_c4438_5567834* | 5D | 546.69 | 9.00E-05 | 11.63 | 2018 | C |
| *wsnp_JD_c4438_5567834* | 5D | 546.69 | 4.10E-04 | 9.33 | 2017 | C |
| *BobWhite_c4438_162* | 5D | 546.69 | 7.00E-06 | 14.18 | 2018 | G |
| *BobWhite_c4438_162* | 5D | 546.69 | 2.50E-04 | 8.89 | 2017 | G |
| *IACX10520* | 5D | 546.69 | 2.00E-05 | 11.63 | 2018 | T |
| *IACX10520* | 5D | 546.69 | 1.10E-04 | 9.33 | 2017 | T |
| *BS00088587_51* | 5D | 546.69 | 2.00E-05 | 11.63 | 2018 | G |
| *BS00088587_51* | 5D | 546.69 | 1.10E-04 | 9.33 | 2017 | G |
| *D_GDS7LZN01CBWNE_99* | 5D | 546.7 | 2.00E-05 | 11.63 | 2018 | T |
| *D_GDS7LZN01CBWNE_99* | 5D | 546.7 | 1.10E-04 | 9.33 | 2017 | T |
| *Kukri_c5528_603* | 5D | 546.7 | 2.00E-05 | 11.63 | 2018 | C |
| *Kukri_c5528_603* | 5D | 546.7 | 1.10E-04 | 9.33 | 2017 | C |
| *Excalibur_c42190_383* | 5D | 546.91 | 4.10E-04 | 9.57 | 2018 | A |
| *Excalibur_c42190_383* | 5D | 546.91 | 4.40E-04 | 9.55 | 2017 | A |
| *Excalibur_c28592_377* | 5D | 546.91 | 9.00E-05 | 9.47 | 2018 | T |
| *Excalibur_c28592_377* | 5D | 546.91 | 3.00E-04 | 8.11 | 2017 | T |
| *Excalibur_c28592_173* | 5D | 546.91 | 4.10E-04 | 9.57 | 2018 | T |
| *Excalibur_c28592_173* | 5D | 546.91 | 4.40E-04 | 9.55 | 2017 | T |
| *Excalibur_c14043_548* | 5D | 546.91 | 9.00E-05 | 9.47 | 2018 | C |
| *Excalibur_c14043_548* | 5D | 546.91 | 3.00E-04 | 8.11 | 2017 | C |
| *CAP8_c145_89* | 5D | 547.27 | 2.00E-05 | 11.63 | 2018 | T |
| *CAP8_c145_89* | 5D | 547.27 | 1.10E-04 | 9.33 | 2017 | T |
| *wsnp_CAP11_c209_198467* | 5D | 547.27 | 2.00E-05 | 11.65 | 2018 | A |
| *wsnp_CAP11_c209_198467* | 5D | 547.27 | 1.30E-04 | 9.37 | 2017 | A |
| *BS00011794_51* | 5D | 547.27 | 2.00E-05 | 11.65 | 2018 | T |
| *BS00011794_51* | 5D | 547.27 | 1.30E-04 | 9.37 | 2017 | T |
| *wsnp_CAP11_c209_198671* | 5D | 547.27 | 3.08E-04 | 11.3 | 2018 | T |
| *BS00022036_51* | 5D | 547.27 | 4.53E-04 | 9.21 | 2018 | A |
| *RAC875_c65515_283* | 6B | 119.13 | 3.69E-05 | 10.98 | 2018 | T |
| *Kukri_c609_328* | 6B | 691.83 | 3.23E-05 | 10.82 | 2018 | A |
| *BobWhite_c22875_239* | 7A | 661.3 | 2.20E-04 | 8.53 | 2018 | C |
| *BobWhite_c22875_239* | 7A | 661.3 | 3.00E-04 | 8.12 | 2017 | C |
| *BobWhite_rep_c52876_72* | 7B | 612.26 | 1.60E-04 | 8.98 | 2017 | G |
| *Ex_c38233_456* | 7B | 743.66 | 2.38E-04 | 8.48 | 2018 | C |

a Markers were detected at the threshold -log10 (P) = 3.0.

b Physical positions of SNP markers based on wheat genome sequences from the International Wheat Genome Sequencing Consortium (IWGSC, http://www.wheatgenome.org/).

c Percentage of phenotypic variance explained by the MTA.

**Additional file 3: Table S3** The cultivars belonging to different haplotype and their FHB severities

| **Name** | **2017PSS** | **2018PSS** | **Mean** | **Haplotype^a^** |
| --- | --- | --- | --- | --- |
| 2-SL76 | 87.21 | 77.34 | 82.27 | 1 |
| 4-Bobwhite | 29.72 | 37.09 | 33.40 | 1 |
| 5-H35 | 71.50 | 71.89 | 71.70 | 1 |
| 6-K35 | 44.12 | 51.02 | 47.57 | 1 |
| 7-NR98117-20S | 83.00 | 83.25 | 83.13 | 1 |
| 8-N553 | 11.79 | 16.43 | 14.11 | 1 |
| 9-AI73 | 22.52 | 15.20 | 18.86 | 1 |
| 11-ANNONG0721 | 48.53 | 78.61 | 63.57 | 1 |
| 13-ANNONG8455 | 70.00 | 47.87 | 58.93 | 1 |
| 14-ANNONG92484 | 44.16 | 66.67 | 55.41 | 1 |
| 15-BAIQUAN565 | 85.29 | 78.11 | 81.70 | 1 |
| 17-BEIJING11 | 15.34 | 24.21 | 19.78 | 1 |
| 18-BEIJING12 | 72.43 | 75.86 | 74.15 | 1 |
| 20-BIMAI6 | 70.62 | 96.95 | 83.79 | 1 |
| 21-BIMA1 | 66.49 | 89.20 | 77.84 | 1 |
| 22-BIMA4 | 66.18 | 97.93 | 82.05 | 1 |
| 23-BOAI7023 | 36.76 | 65.09 | 50.93 | 1 |
| 24-CHUAN78001 | 22.82 | 44.61 | 33.71 | 1 |
| 25-CHUAN80-466 | 28.34 | 58.42 | 43.38 | 1 |
| 26-CHUAN9920 | 50.00 | 34.40 | 42.20 | 1 |
| 27-CHUANMAI37 | 27.75 | 9.91 | 18.83 | 1 |
| 28-CHUANMAI41 | 13.47 | 9.00 | 11.24 | 1 |
| 30-CHUANYU16 | 13.24 | 29.68 | 21.46 | 1 |
| 31-CHUANYU21526 | 44.39 | 43.24 | 43.81 | 1 |
| 32-E1161 | 30.43 | 67.14 | 48.79 | 1 |
| 33-EMAI9 | 50.51 | 12.42 | 31.47 | 1 |
| 34-FAZHAN5 | 40.59 | 44.50 | 42.55 | 1 |
| 35-FUFAN17 | 22.71 | 29.41 | 26.06 | 1 |
| 36-FUMIAN2 | 36.67 | 66.81 | 51.74 | 1 |
| 37-GAN83 | 34.48 | 33.01 | 33.75 | 1 |
| 38-GANMAI7 | 46.11 | 40.91 | 43.51 | 1 |
| 39-ZHENMAI4 | 45.35 | 46.59 | 45.97 | 1 |
| 40-GAOCHENG8901 | 86.96 | 47.14 | 67.05 | 1 |
| 41-HAINING75-39 | 28.57 | 69.15 | 48.86 | 1 |
| 43-HECHUN12 | 56.99 | 66.34 | 61.67 | 1 |
| 44-HENONG822 | 42.86 | 29.76 | 36.31 | 1 |
| 47-HUAPEI128-8 | 72.65 | 45.21 | 58.93 | 1 |
| 48-HUAPEI128BAIDA | 41.31 | 41.15 | 41.23 | 1 |
| 51-JIMAI20 | 74.19 | 94.06 | 84.13 | 1 |
| 52-JINAN14 | 94.68 | 82.35 | 88.52 | 1 |
| 53-JINAN16 | 90.24 | 63.50 | 76.87 | 1 |
| 54-JINAN8 | 56.86 | 88.79 | 72.82 | 1 |
| 55-JIMAI24 | 45.96 | 63.29 | 54.62 | 1 |
| 57-JINGMAI33 | 59.57 | 51.49 | 55.53 | 1 |
| 58-JINGMAI52 | 46.35 | 61.62 | 53.99 | 1 |
| 61-JINGZHOU1 | 88.33 | 66.18 | 77.26 | 1 |
| 63-JINGZHOU66 | 47.21 | 64.50 | 55.85 | 1 |
| 64-KAIFENG10 | 94.42 | 27.33 | 60.87 | 1 |
| 65-KECHUN5 | 71.43 | 75.69 | 73.56 | 1 |
| 67-LINMAI2 | 73.08 | 80.93 | 77.00 | 1 |
| 72-LUMAI2 | 96.74 | 85.42 | 91.08 | 1 |
| 73-LUMAI3 | 31.98 | 57.87 | 44.92 | 1 |
| 77-MIANNONG4 | 74.50 | 88.21 | 81.35 | 1 |
| 78-MIANYANG15 | 69.68 | 65.30 | 67.49 | 1 |
| 79-MIANYANG30 | 46.51 | 63.27 | 54.89 | 1 |
| 80-MIANYANG31 | 39.34 | 24.51 | 31.93 | 1 |
| 81-MOBA66 | 90.45 | 75.59 | 83.02 | 1 |
| 83-NINGAI8628 | 34.34 | 56.50 | 45.42 | 1 |
| 84-NINGMAI10 | 6.88 | 17.24 | 12.06 | 1 |
| 85-NINGMAI11 | 9.55 | 5.86 | 7.71 | 1 |
| 86-NINGMAI12 | 86.46 | 37.98 | 62.22 | 1 |
| 89-NINGMAI3 | 29.67 | 25.23 | 27.45 | 1 |
| 90-NINGMAI6 | 30.57 | 27.54 | 29.05 | 1 |
| 92-NINGMAI8 | 44.44 | 41.99 | 43.22 | 1 |
| 95-ZHONGLUOTIEGAN | 33.90 | 25.16 | 29.53 | 1 |
| 97-NONGDA116 | 65.85 | 63.90 | 64.88 | 1 |
| 101-PUBIN04-3507 | 91.62 | 83.51 | 87.56 | 1 |
| 103-PUBIN4302AN | 43.78 | 79.21 | 61.49 | 1 |
| 104-PUBIN4313AN | 25.23 | 49.03 | 37.13 | 1 |
| 106-QIANHUAN3 | 82.81 | 49.06 | 65.93 | 1 |
| 109-SHANNONGFU63 | 52.15 | 56.38 | 54.27 | 1 |
| 110-SHAN253 | 36.65 | 49.25 | 42.95 | 1 |
| 111-SHAN76 | 21.84 | 94.61 | 58.22 | 1 |
| 112-SHAN7859 | 48.07 | 83.42 | 65.74 | 1 |
| 113-SHAN8242 | 36.65 | 68.90 | 52.77 | 1 |
| 114-SHANMAI150 | 20.49 | 63.33 | 41.91 | 1 |
| 115-SHANNONG17 | 68.04 | 73.98 | 71.01 | 1 |
| 116-SHANNONG21 | 52.86 | 46.19 | 49.52 | 1 |
| 122-SHIMAI12 | 53.85 | 50.91 | 52.38 | 1 |
| 125-SUMAI2 | 11.89 | 26.96 | 19.43 | 1 |
| 128-SUINONG3 | 97.00 | 70.94 | 83.97 | 1 |
| 131-TAIYUAN567 | 51.39 | 35.65 | 43.52 | 1 |
| 132-WANMAI17 | 12.29 | 54.55 | 33.42 | 1 |
| 133-WANMAI18 | 58.12 | 60.20 | 59.16 | 1 |
| 134-WANMAI32 | 23.78 | 44.78 | 34.28 | 1 |
| 135-WANMAI47 | 95.21 | 76.00 | 85.61 | 1 |
| 136-WANMAI48 | 35.65 | 41.23 | 38.44 | 1 |
| 138-WANXI7751 | 50.56 | 37.89 | 44.22 | 1 |
| 142-WENMAI8 | 85.22 | 83.02 | 84.12 | 1 |
| 145-XIANSHIXIN | 21.56 | 26.12 | 23.84 | 1 |
| 146-XINONG2611 | 65.38 | 18.89 | 42.14 | 1 |
| 147-XIANG1437 | 35.24 | 52.58 | 43.91 | 1 |
| 149-XIANG1479 | 56.76 | 52.49 | 54.62 | 1 |
| 150-XIANG673-1 | 49.24 | 52.17 | 50.71 | 1 |
| 152-XIANG1599 | 34.45 | 62.96 | 48.71 | 1 |
| 153-XIANGIRWAN | 21.69 | 23.62 | 22.66 | 1 |
| 156-XIANGNONG7984 | 65.92 | 42.67 | 54.30 | 1 |
| 160-XIANGMAI48 | 55.61 | 81.19 | 68.40 | 1 |
| 161-XIANGMAI5 | 32.84 | 50.71 | 41.77 | 1 |
| 162-XIAOYAN168 | 27.57 | 24.27 | 25.92 | 1 |
| 163-XIAOYAN54 | 11.35 | 15.71 | 13.53 | 1 |
| 164-XIAOYAN5 | 64.73 | 93.53 | 79.13 | 1 |
| 166-XIAOYAN96 | 87.68 | 38.86 | 63.27 | 1 |
| 167-SUDI8112 | 30.23 | 25.33 | 27.78 | 1 |
| 168-XUMAI27 | 88.52 | 100.00 | 94.26 | 1 |
| 169-XUZHOU15 | 40.63 | 40.37 | 40.50 | 1 |
| 170-XUZHOU20 | 92.57 | 58.5 | 75.535 | 1 |
| 172-XUZHOU8785 | 32.65 | 77.45 | 55.05 | 1 |
| 174-XUAN7 | 67.15 | 84.54 | 75.85 | 1 |
| 175-YANFU188 | 92.79 | 89.27 | 91.03 | 1 |
| 177-YANDA25 | 10.92 | 62.38 | 36.65 | 1 |
| 178-YANSHI4 | 29.28 | 40.50 | 34.89 | 1 |
| 180-YANGFUMAI2 | 7.45 | 34.39 | 20.92 | 1 |
| 181-YANGFUMAI4 | 21.28 | 36.54 | 28.91 | 1 |
| 182-YANGFUMAI5242 | 18.48 | 29.25 | 23.86 | 1 |
| 183-YANGMAI10 | 18.09 | 19.90 | 19.00 | 1 |
| 184-YANGMAI11 | 18.13 | 16.56 | 17.35 | 1 |
| 185-YANGMAI13 | 55.33 | 51.28 | 53.31 | 1 |
| 186-YANGMAI14 | 10.81 | 7.65 | 9.23 | 1 |
| 187-YANGMAI16 | 10.40 | 23.53 | 16.96 | 1 |
| 189-YANGMAI19 | 7.95 | 13.27 | 10.61 | 1 |
| 191-YANGMAI2 | 30.69 | 21.35 | 26.02 | 1 |
| 192-YANGMAI3 | 29.79 | 22.95 | 26.37 | 1 |
| 193-YANGMAI6 | 6.80 | 20.10 | 13.45 | 1 |
| 194-YANGMAI9 | 17.73 | 35.68 | 26.71 | 1 |
| 197-YOUMANGBAI4 | 58.82 | 76.53 | 67.67 | 1 |
| 198-YOUMANGHONG18 | 75.66 | 57.95 | 66.81 | 1 |
| 200-YU30691-3 | 33.99 | 47.87 | 40.93 | 1 |
| 201-YU30691-6 | 32.26 | 57.14 | 44.70 | 1 |
| 203-YU85-2325 | 47.87 | 70.10 | 58.99 | 1 |
| 204-YUBAO11 | 31.16 | 50.00 | 40.58 | 1 |
| 205-YUMAI10 | 22.35 | 51.28 | 36.81 | 1 |
| 206-YUMAI13 | 56.22 | 55.41 | 55.81 | 1 |
| 208-YUMAI17 | 29.86 | 22.40 | 26.13 | 1 |
| 209-YUMAI50 | 86.08 | 62.56 | 74.32 | 1 |
| 212-YUNMAI27 | 70.42 | 62.98 | 66.70 | 1 |
| 214-ZANG2726 | 12.56 | 59.19 | 35.88 | 1 |
| 215-ZHEFENG2 | 42.16 | 51.71 | 46.93 | 1 |
| 216-ZHENONGDA85PIN8 | 18.23 | 19.38 | 18.80 | 1 |
| 219-ZHEN7495 | 26.23 | 21.82 | 24.02 | 1 |
| 220-ZHEN7630 | 54.59 | 51.64 | 53.12 | 1 |
| 221-ZHENMAI1 | 31.22 | 54.17 | 42.69 | 1 |
| 223-ZHENMAI6 | 6.38 | 10.88 | 8.63 | 1 |
| 225-ZHENG87302 | 36.32 | 24.33 | 30.32 | 1 |
| 226-ZHENGMAI004 | 95.52 | 93.93 | 94.72 | 1 |
| 227-ZHENGYIN1 | 13.92 | 37.80 | 25.86 | 1 |
| 231-ZHENGZHOU741 | 61.88 | 78.70 | 70.29 | 1 |
| 232-ZHENGZHOU9285 | 11.65 | 15.42 | 13.53 | 1 |
| 234-ZHOUYOU102 | 52.76 | 69.63 | 61.19 | 1 |
| 29-XIFENG | 10.94 | 28.16 | 19.55 | 2 |
| 87-NINGMAI13 | 11.87 | 16.97 | 14.42 | 2 |
| 93-NINGMAI9 | 24.73 | 32.99 | 28.86 | 2 |
| 94-NINGMAI18 | 7.30 | 23.95 | 15.63 | 2 |
| 105-QIANHUAN2 | 49.46 | 33.04 | 41.25 | 2 |
| 126-SUMAI5 | 15.96 | 7.35 | 11.65 | 2 |
| 137-WANPIN8056 | 39.91 | 33.63 | 36.77 | 2 |
| 143-WU78 | 12.95 | 12.73 | 12.84 | 2 |
| 151-XIANG675-2 | 12.97 | 38.58 | 25.78 | 2 |
| 154-XIANG10 | 7.07 | 15.60 | 11.34 | 2 |
| 155-XIANGNONG153 | 9.36 | 16.55 | 12.96 | 2 |
| 157-XIANGNONG13744 | 4.42 | 4.15 | 4.29 | 2 |
| 159-XIANGNONG4053 | 19.30 | 45.74 | 32.52 | 2 |
| 176-YANNONG19 | 45.11 | 36.88 | 41.00 | 2 |
| 179-YANG85-85 | 23.47 | 26.63 | 25.05 | 2 |
| 188-YANGMAI18 | 10.38 | 10.00 | 10.19 | 2 |
| 190-YANGMAI1 | 13.88 | 7.18 | 10.53 | 2 |
| 222-ZHENMAI5 | 5.31 | 16.88 | 11.10 | 2 |
| 233-ZHONGYOU16 | 9.44 | 16.83 | 13.14 | 2 |
| 127-SUMAI6 | 16.85 | 16.15 | 16.50 | 3 |
| 217-ZHENMAI168 | 19.27 | 13.89 | 16.58 | 3 |
| 218-ZHENMAI9 | 6.77 | 9.55 | 8.16 | 3 |

a Three haplotype groups revealed through haplotype analyses of the associated markers

**Additional file 4: Table S4 Physical positions of reported FHB resistance QTL related to those in the current study**

| **Chr.** | **Source** | **QTL/Gene** | **Linked marker** | **Position^a^ (Mb)** | **Reference** |
| --- | --- | --- | --- | --- | --- |
| 1B | Huangfangzhu |  | *Xbarc207* | 506.33 | Li et al. 2012 |
| 1B | Wangshuibai |  | *Xwms759* | unknown | Zhou et al. 2004 |
| 1B | Wangshuibai |  | *Xbarc302* | 455.99 | Liu et al. 2009 |
|  |  |  | *Xglu-1B* | unknown |  |
| 1B | Seri 82 |  | *Xgwm273* | 212.51 | Liu et al. 2009 |
|  |  |  | *Xbarc302* | 455.99 |  |
| 1B | CM 82036 |  | *Xbarc302* | 455.99 | Liu et al. 2009 |
|  |  |  | *Xgwm153* | 628.83 |  |
| 1B | Fundulea201R |  | *Xgwm264* | 15.03 | Liu et al. 2009 |
|  |  |  | *Xgwm131* | 459.16 |  |
| 1B | Romanus |  | *Xgwm018* | 222.58 | Liu et al. 2009 |
|  |  |  | *Xgwm153* | 628.83 |  |
| 1B | Arina |  | *Xgwm264* | 15.03 | Liu et al. 2009 |
|  |  |  | *Xgwm273* | 212.51 |  |
| 1B | Ralito |  | *Xgwm018* | 222.58 | Liu et al. 2009 |
|  |  |  | *Xgwm131* | 459.16 |  |
| 1B | Yumechikara |  | *Glu-B3* | unknown | Nishio et al. 2016 |
|  |  |  | *Xbarc32* | unknown |  |
| 4AL | Arina | *QFhs.fal-4AL* | *Xgwm160* | 357.2 | Paillard et al. 2004 |
|  |  |  | *Xcdo545* | unknown |  |
| 4AL | Pirat |  | *XP7553-254.AR* | unknown | Holzapfel et al. 2008 |
| 4AL | Apache |  | *XP7452-646* | unknown | Holzapfel et al. 2008 |
| 5D | Chokwang | *Qfhb.ksu-5DL1* | *Xbarc239* | 420.96 | Yang et al. 2005 |
| 5D | Alondra‘s’ |  | *Xgwm190* | unknown | Jia et al. 2005 |
|  |  |  | *Xgwm358* | 120.61 |  |
| 7A | Huangfangzhu | *Qfhb.uhgl-7AL* | *Xgwm276* | 642.88 | Li et al. 2012 |
|  |  |  | *Xbarc121* | 611.84 |  |
| 7A | Wangshuibai |  | *Xgwm276* | 642.88 | Jia et al. 2005 |
|  |  |  | *Xgwm282* | 681.39 |  |
| 7A | Romanus |  | *Xbarc121* | 611.84 | Liu et al. 2009 |
|  |  |  | *Xbarc29* | 616.22 |  |
| 7A | NK93604 |  | *Xbarc29* | 616.22 | Liu et al. 2009 |
|  |  |  | *Xgwm276* | 642.88 |  |
| 7A | Spark |  | *Xbarc121* | 611.84 | Liu et al. 2009 |
|  |  |  | *Xgwm276* | 642.88 |  |
| 7A | Fontana |  | *Xfbb121* | 611.84 | Liu et al. 2009 |
|  |  |  | *Xgwm233* | 11.36 |  |
| 7A | Wangshuibai |  | *Xwms1083* | unknown | Zhou et al. 2004 |

^a^ Physical positions of SNP markers based on wheat genome sequences from the International Wheat Genome Sequencing Consortium (IWGSC, http://www.wheatgenome.org/).

| **Additional file 5: Table S5** The regions associated with FHB resistance with the same function exists in the corresponding sections of 4A and 5D | | | | | | |
| --- | --- | --- | --- | --- | --- | --- |
| **No** | **Pos^a^ (4A)** | **Transcript^b^ 4A** | **Transcript^b^ 5D** | **Pos^a^(5D)** | **Predicted function^c^** | **Gene** |
| 1 | 621,793,154-621,793,513 | *TraesCS4A02G341200* | - | - | uncharacterized protein LOC109785378 | *LOC109785378* |
| 2 | 621,796,765-621,806,512 | *TraesCS4A02G341300* | - | - | 4-hydroxyphenylacetaldehyde oxime monooxygenase-like | *LOC109733661* |
| 3 | 621,813,067-621,816,042 | *TraesCS4A02G341500* | *TraesCS5D02G531400* | 546,921,823-546,924,651 | GDSL esterase/lipase At3g48460 | *LOC109767068* |
| 4 | 621,816,065-621,819,950 | *TraesCS4A02G341600* | *TraesCS5D02G531300* | 546,917,879-546,922,510 | Serine/threonine protein phosphatase 2A 59 kDa regulatory subunit B' gamma isoform | *TRIUR3_02855* |
| 5 | 621,841,671-621,849,555 | *TraesCS4A02G341700* | *TraesCS5D02G531200* | 546,906,214-546,913,767 | PTI1-like tyrosine-protein kinase 1 | *LOC109767070* |
| 6 | 622,120,855-622,125,004 | *TraesCS4A02G341800* | *-* | - | probable thiol methyltransferase 2 | *LOC109760710* |
| 7 | 622,126,363-622,133,721 | *TraesCS4A02G341900* | *TraesCS5D02G531100* | 546,858,080-546,865,594 | Zinc finger MYND domain-containing protein 15 | *TRIUR3_23753* |
| 8 | 622,162,368-622,162,771 | *TraesCS4A02G342000* | *-* | - | hypothetical protein TRIUR3_00914 | *TRIUR3_00914* |
| 9 | 622,173,126-622,175,400 | *TraesCS4A02G342100* | *TraesCS5D02G531000* | 546,825,426-546,827,787 | pentatricopeptide repeat-containing protein At2g27610-like | *LOC109760711* |
| 10 | 622,175,583-622,180,034 | *TraesCS4A02G342200* | *TraesCS5D02G530900* | 546,820,679-546,825,262 | poly(ADP-ribose) glycohydrolase 1-like | *LOC109760709* |
| 11 | 622,180,065-622,181,173 | *TraesCS4A02G342300* | *TraesCS5D02G530800* | 546,819,301-546,820,725 | probable thiol methyltransferase 2 | *LOC109760710* |
| 12 | 622,198,854-622,203,554 | *TraesCS4A02G507700LC* | *-* | - | protein FAR1-RELATED SEQUENCE 6-like | *LOC109763821* |
| 13 | 622,221,038-622,227,952 | *TraesCS4A02G342400* | *TraesCS5D02G530700* | 546,695,539-546,703,266 | probable ion channel CASTOR isoform X2 | *LOC109763818* |
| 14 | [622,235,357-622,238,103](http://plants.ensembl.org/Triticum_aestivum/Location/View?db=core;g=TraesCS4A02G342500;r=4A:622235357-622238103;t=TraesCS4A02G342500.1) | *TraesCS4A02G342500* | *TraesCS5D02G530600* | 546,687,753-546,690,173 | Putative receptor protein kinase ZmPK1 | *TRIUR3_00921* |
| 15 | 622,275,349-622,277,620 | *TraesCS4A02G342600* | *TraesCS5D02G530400* | 546,678,820-546,681,068 | hypothetical protein TRIUR3_00922 | *TRIUR3_00922* |
| 16 | 622,281,717-622,287,276 | *TraesCS4A02G342700* | *TraesCS5D02G530300* | 546,651,779-546,657,365 | putative RNA-binding protein Luc7-like 2 | *LOC109763817* |
| 17 | 622,288,319-622,290,370 | *TraesCS4A02G342800* | *TraesCS5D02G530200* | 546,566,054-546,567,836 | 30S ribosomal protein S6 alpha, chloroplastic | *LOC109763823* |
| 18 | 622,439,970-622,441,062 | *TraesCS4A02G342900* | *-* | - | tropinone reductase homolog At2g29370-like | *LOC109763826* |
| 19 | 622,484,486-622,485,103 | *TraesCS4A02G343000* | *TraesCS5D02G530000* | 546,454,980-546,455,603 | hypothetical protein BRADI_4g42460v3 | *BRADI_4g42460v3* |
| 20 | 622,509,024-622,510,602 | *TraesCS4A02G343100* | *-* | - | tropinone reductase homolog At2g29370-like [Aegilops tauschii subsp. tauschii] | *LOC109763826* |

a Physical positions based on wheat genome sequences from the International Wheat Genome Sequencing Consortium (IWGSC, http://www.wheatgenome.org/).

b *T. aestivum* gene transcripts and their domains were explored in Ensembl (using the transcript table link).

c The sequences of *T.aestivum* gene were blasted in the NCBI (http://www.ncbi.nlm.nih.gov/), databases to identify putative gene functions.
